# Development of an Operational Protocol for Animal Hoarding: A Conceptual Proposal Based on Multidisciplinary Field Experience

**DOI:** 10.3390/ani15213222

**Published:** 2025-11-06

**Authors:** Francesca Bellini, Alberto Cal, Alessia Liverini, Gianna Regoli, Giancarlo Ruffo

**Affiliations:** 1 Local Health Authority Rome 1, 00193 Rome, Italy; francesca.bellini@aslroma1.it; 2 Private Practice, 00137 Rome, Italy; albertocal.medvet@gmail.com; 3 Local Health Authority Rome 4, 00053 Civitavecchia, Italy; alessia.liverini@aslroma4.it (A.L.); gianna.regoli@aslroma4.it (G.R.); 4 Department of Veterinary Medicine and Animal Sciences, University of Milan, 26900 Lodi, Italy

**Keywords:** animal hoarding, animal welfare, public health, human psychological distress, operational protocol

## Abstract

Animal hoarding is a complex and multifactorial phenomenon that poses serious risks to animal welfare, public health, and environmental balance. Despite increasing attention, current interventions often remain fragmented and lack integration across medical, psychological, and social domains. Based on a critical review of existing tools and field experience in the Italian context, this study proposes a structured operational protocol to support multidisciplinary teams in the assessment and management of animal hoarding cases. The protocol integrates three complementary tools: a preliminary observational form, a clinical-relational interview, and a veterinary health form. Designed to be modular, replicable, and shareable among professionals from diverse backgrounds, the protocol aims to promote a One Welfare approach, recognizing the systemic interconnection between animal suffering, human psychological distress, and environmental degradation. While further experimental validation is required, this conceptual model provides a concrete operational basis for structured interventions and consistent data collection in support of research and public health.

## Simple Summary

Animal hoarding is a complex and often under-recognized form of neglect, characterized by the accumulation of a large number of animals without ensuring adequate care, hygiene, or living conditions. This study underscores the need for a structured and multidisciplinary approach that integrates veterinary, psychological, and social perspectives. Drawing on field experience and literature review, we propose an operational framework for the assessment and management of animal hoarding cases, emphasizing the importance of early intervention and coordinated professional collaboration to protect both human and animal welfare.

## 1. Introduction

Animal hoarding (AH) is a complex condition with profound implications for animal welfare, public and mental health, and environmental safety [1]. Although internationally recognized as a severe manifestation of Hoarding Disorder (HD), as defined in the DSM-5 [2], it represents a global issue documented across diverse cultural and socio-economic contexts. However, the phenomenon remains largely overlooked in Italy and lacks a systematic framework, despite a steady increase in reports from health services and public safety authorities.

The literature has documented the main psychopathological features of AH, as well as its environmental and health-related consequences [3,4,5]. However, shared tools are still lacking for collecting and connecting relevant information—ranging from the living conditions of animals and the environment, to the subjective experiences of the individuals involved, to the social impact and post-intervention outcomes [6]. Crucial data—such as the fate of removed animals, the evolution of living conditions, the long-term effectiveness of interventions, and recurrence rates—remain fragmented or entirely unavailable [7,8]. In many cases, denial of the problem and dysfunctional attachment to animals hinder effective care and intervention efforts [9].

The Italian operational context involves numerous stakeholders—veterinary services, mental health services, law enforcement, social services, and public health authorities—who often lack structured coordination [10]. In this scenario, there is an urgent need for tools that can overcome the current fragmentation and enable an integrated understanding of the phenomenon, guiding assessments, interventions, and follow-up in alignment with the regulatory framework.

This study proposes a conceptual methodological framework for managing animal hoarding (AH) cases, developed through the analysis of critical issues encountered during the application of an adapted observational tool, the Case Report Form [10], to 48 documented cases in the Lazio region between 2019 and 2024. The proposed model integrates objective assessments of environmental and veterinary conditions with psychological and relational exploration tools, which are currently lacking in existing approaches.

Although grounded in theoretical foundations and tested through simulation, the protocol represents a well-structured and rational preliminary model designed to address current methodological gaps in animal hoarding management. It serves as a starting point for the development of a validated tool that will require verification in terms of reliability, inter-rater consistency, and practical applicability in real-world settings.

The following sections outline the legal and psychopathological context that forms the theoretical and operational basis of this framework.

## 2. Legal Framework of the Phenomenon in Italy

Although animal hoarding is clinically recognized as a manifestation of Hoarding Disorder in the DSM-5, it entails significant legal consequences in the Italian context. The behaviors exhibited by hoarders—while often rooted in a compromised psychiatric condition—do not exempt individuals from legal responsibility: under current legislation, various criminal and administrative offenses can be identified, even in the absence of intent [11]. The consequences affect animal welfare, environmental hygiene, public health, and social coexistence. This section provides an overview of the main legal instruments available and the operational challenges encountered in their application.

### 2.1. Criminal and Administrative Framework in Italian Legislation

To date, no specific legislation on animal hoarding exists in Italy. Nevertheless, such cases can be prosecuted under a combination of existing legal provisions, including the following:

Article 544-ter of the Italian Penal Code—Cruelty to animals, punishing acts that cause unnecessary suffering or injury;

Article 727 of the Italian Penal Code—Keeping animals in conditions incompatible with their nature, including neglect and abandonment;

Article 544-sexies of the Italian Penal Code—Mandatory confiscation of animals upon conviction;

Article 650 of the Italian Penal Code—Failure to comply with orders issued by public authorities;

Article 674 of the Italian Penal Code—Emissions that are dangerous or cause public nuisance;

Legislative Decree No. 134/2022—Establishes the National Animal Identification and Traceability System (SINAC);

Legislative Decree No. 152/2006 and Royal Decree No. 1265/1934—Environmental and public health protection regulations;

Municipal regulations—Include limits on the number of animals, hygiene standards, and mandatory registration requirements.

#### 2.1.1. Keeping Animals in Incompatible Conditions (Article 727 of the Italian Penal Code)

In Italy, keeping animals in inadequate conditions may constitute a criminal offense under Article 727 of the Italian Penal Code, which penalizes anyone who keeps animals in conditions “incompatible with their nature” and likely to cause severe suffering. This includes overcrowding, poor hygiene, inadequate shelter, and the lack of essential care such as food, water, and veterinary assistance. Applicable sanctions include imprisonment for up to one year or a fine of up to €10,000. This provision applies even in the absence of intent—for example, when the number of animals exceeds the individual’s ability to ensure proper care, cleanliness, and management. These conditions are commonly observed in animal hoarding cases, even without evidence of deliberate cruelty [11,12].

Italian case law has repeatedly confirmed the applicability of Article 727 in hoarding contexts, establishing criminal liability when animals are found in overcrowded, unsanitary environments and deprived of basic needs. Notable rulings by the Italian Supreme Court (No. 44287/2007; No. 49298/2012; No. 1510/2019) have been instrumental in clarifying the legal interpretation of animal hoarding as a punishable offense under Article 727, even in the absence of overt physical violence.

#### 2.1.2. Animal Cruelty (Article 544-Ter of the Italian Penal Code)

Severe cases of pathological accumulation may constitute the criminal offense of animal cruelty, as defined in Article 544-ter of the Italian Penal Code. This provision, recently amended, punishes with imprisonment from six months to two years and a fine ranging from €5000 to €30,000 anyone who, “out of cruelty or without necessity,” causes injury to an animal, or subjects it to abuse or to conditions that seriously compromise its health or well-being. More severe penalties apply if the animal dies or if the act involves the administration of prohibited substances.

In the context of animal hoarding, cruelty does not necessarily take the form of violent acts but may emerge as chronic neglect, overcrowding, lack of veterinary care, insufficient space, and severely unhygienic conditions [13]. Taken together, these factors may lead to physical and psychological suffering severe enough to meet the threshold for criminal liability [14].

A landmark ruling by the Court of Appeal of Turin (11 April 2014) upheld the conviction of a woman who kept 81 dogs in a state of severe degradation, recognizing that prolonged neglect constitutes a form of cruelty in itself. More recently, the Italian Supreme Court (Third Criminal Division, Judgment No. 15453/2023) ruled that physical injury is not required: merely exposing animals to severely compromised living conditions may constitute a criminal offense, clearly distinguishing it from the lesser administrative violation under Article 727.

### 2.2. Additional Criminal and Administrative Measures

#### 2.2.1. Noncompliance with Official Orders (Article 650 of the Italian Penal Code)

One of the most challenging aspects of animal hoarding is the high rate of recidivism [15,16]. It is not uncommon for individuals to continue keeping animals even after seizures or criminal convictions, systematically evading the restrictions imposed by competent authorities [8,9]. When a person ignores mayoral orders, health authority directives, or judicial rulings—for example, by refusing access to the premises, introducing new animals, or failing to implement mandated measures—this may constitute a criminal offense under Article 650 of the Italian Penal Code. This provision penalizes noncompliance with legally issued measures adopted for reasons of justice, public safety, or hygiene.

A particularly notable case occurred in a province of the Lazio region, where a woman previously convicted of animal cruelty continued to accumulate animals despite multiple prohibitory orders issued against her [10]. Her repeated violations of a public health ordinance led to charges under Article 650, highlighting the urgent need for more effective legal tools to prevent chronic noncompliance and recurrent behavior.

#### 2.2.2. Mandatory Confiscation and Its Limitations (Article 544-Sexies of the Italian Penal Code)

Article 544-sexies of the Italian Penal Code, introduced by Law No. 189/2004, provides for the mandatory confiscation of animals in cases of conviction for mistreatment (Article 544-ter) or for keeping animals in conditions incompatible with their nature (Article 727). This measure aims to prevent animals from remaining in the custody of individuals deemed criminally responsible for their suffering.

However, recent case law has highlighted critical issues in the automatic application of this measure. The Court of Cassation (Judgment No. 4463/2022) ruled that, in cases where the offense is extinguished following the successful completion of a probation program, confiscation cannot be imposed, as no final conviction is issued. Consequently, animals may be returned even to individuals who are clearly unfit to care for them.

This legislative gap undermines the effectiveness of interventions and raises concerns about the balance between procedural safeguards and the protection of animal welfare. In response, legal scholars have proposed a series of reforms, including the following:

Permanent bans on animal ownership for individuals with prior convictions;

Harsher penalties for repeat offenders;

Mandatory rehabilitation programs that combine judicial sanctions with psychiatric and social support.

Such programs—already endorsed by several authors [1,16,17]—could help break the cycle of recidivism by offering individuals tools for self-awareness, social reintegration, and alternatives to compulsive hoarding.

### 2.3. Prevention and Control Legislation

#### 2.3.1. Animal Identification and Monitoring (Legislative Decree No. 134/2022)

Legislative Decree No. 134/2022 introduced a strengthened system for the identification, registration, and traceability of companion animals through the establishment of the National Information System for Companion Animals (SINAC). Although not specifically designed to address animal hoarding, this legislation represents a potentially effective preventive tool, as it enables the assignment of individual ownership responsibilities and allows authorities to monitor conditions of overcrowding or disorganized animal management.

Failure to register companion animals constitutes an administrative offense, punishable by fines ranging from €150 to €900 per unregistered animal. In hoarding contexts, systematic non-compliance with this obligation may serve as an early indicator of dysfunctional management and provide objective grounds for timely intervention.

The decree also establishes quantitative thresholds to distinguish private ownership from breeding activity. For instance, a high number of registered reproductive animals associated with a single individual may indicate the presence of an unregulated breeding operation, subject to fines of up to €7500 and possible confiscation of the animals.

From a relapse prevention perspective, several authors have proposed legislative reforms aimed at strengthening long-term oversight mechanisms. These include the introduction of a national registry of known hoarders, the imposition of permanent bans on animal ownership for individuals previously convicted of relevant offenses, and the establishment of harsher penalties for repeated violations of animal welfare legislation. In addition, the implementation of mandatory rehabilitation programs—combining judicial sanctions with psychiatric and social support interventions—has been advocated as a means to interrupt the cycle of recidivism by equipping individuals with tools for increased awareness, social reintegration, and viable alternatives to compulsive hoarding behaviors [18,19].

#### 2.3.2. Municipal Regulations and Regulatory Disparities

In addition to national legislation, many Italian municipalities have enacted local regulations concerning animal ownership, welfare, and urban hygiene. These provisions typically establish numerical limits, structural requirements, and minimum hygiene standards, along with obligations for registration and inspections conducted by public veterinary services.

However, the lack of uniformity among local regulations poses a significant obstacle to the consistent application of preventive measures. For instance, the Municipality of Rome does not impose numerical limits but assesses animal ownership based on sanitary and environmental conditions (Comune di Roma Capitale, 2005). In contrast, cities such as Milan and Verona impose specific thresholds—five dogs or ten cats per household, respectively—exceeding which requires prior authorization (Comune di Milano, 2020; Comune di Verona, 2020).

This regulatory fragmentation results in substantial disparities in the ability of local authorities to detect and contain hoarding cases, particularly in their early stages. The definition of shared thresholds of concern and the implementation of nationally coordinated intervention protocols are therefore urgently needed to overcome the current variability in interpretation and enforcement practices.

Nevertheless, violations of municipal regulations do allow local authorities to adopt urgent measures, including forced entry into premises, removal of animals, and the issuance of mayoral ordinances.

#### 2.3.3. Emergency Ordinances and the Role of Local Authorities

In Italy, the Mayor—acting as the local health authority—holds the power to issue emergency public health measures under Article 50 of Legislative Decree No. 267/2000 (Consolidated Law on Local Authorities) and Article 32 of Law No. 833/1978 (Establishment of the National Health Service) in response to situations that pose a threat to public health.

In the context of animal hoarding, such measures—known as *ordinanze contingibili e urgenti*—serve as critical legal tools for halting severe environmental degradation while awaiting possible judicial proceedings. These interventions—immediately enforceable upon notification—may include the removal of animals, the prohibition of access to premises, environmental sanitation, and, in the most serious cases, referral of the individual to psychiatric services for clinical evaluation and care.

Although their application may vary across jurisdictions, these emergency measures often represent the only effective and timely mechanism for safeguarding both public health and animal welfare when judicial intervention is absent, delayed, or inadequate.

#### 2.3.4. Environmental Degradation and Violations of Sanitary Regulations

Pathological animal hoarding is frequently associated with extreme hygienic and environmental degradation, including strong odors, the presence of carcasses, accumulations of feces, infestations, and biologically hazardous waste [13,14]. Notably, Article 674 of the Italian Penal Code may apply in such contexts, as it penalizes anyone who causes harmful or offensive emissions—including olfactory ones—that pose a risk to public health or disturb the community. The Italian Supreme Court (Ruling No. 45230/2014) confirmed the criminal relevance of such harmful emissions, even in the absence of documented physical harm.

In addition to the Penal Code, the following legislative provisions are applicable:

Legislative Decree No. 152/2006 (Environmental Code), which assigns to mayors the duty to safeguard environmental health and to adopt urgent measures in the presence of health risks;

Royal Decree No. 1265/1934 (Consolidated Health Laws), which authorizes the issuance of health ordinances in cases of unsanitary environments, indecent housing, or compromised hygiene.

A representative case—documented in official technical records and based on the direct experience of one of the authors (in compliance with current privacy regulations)—occurred in 2013 in a semi-rural area of the Lazio region. Although the term “animal hoarding” was not yet in use, the case presented all the features now associated with the disorder: overcrowding, chronic neglect, severe sanitary degradation, and strong denial of the situation. The intervention, carried out jointly by law enforcement, public veterinary services, local police, and public health authorities, led to the seizure of over 26 dogs and the discovery of more than 200 kg of carcasses in an advanced state of decomposition. The surviving animals were found in critical condition, suffering from malnutrition, parasitic infestations, and suspected infectious diseases. During the inspection, the individual involved stated that they were unable to part even with the deceased animals due to an intense emotional bond. This statement poignantly illustrates the mechanisms of denial and emotional distortion commonly observed in cases of animal hoarding, as widely described in the scientific literature [1,6,20]. The operation concluded with the sanitation of the premises and the initiation of judicial proceedings.

#### 2.3.5. Impact on Neighbors and the Community

Animal hoarding compromises not only the welfare of the animals and the health of the individual involved, but also has significant repercussions for the surrounding community. Residents living nearby frequently report foul odors, persistent noise, infestation risks, and concerns about their own health and safety. These conditions often generate discomfort, neighbor conflicts, and formal requests for intervention by local authorities [21,22].

Such reports typically trigger the involvement of public veterinary services, municipal administrations, law enforcement, and environmental health agencies. In response, some municipalities—including Bologna—have enacted specific regulations requiring owners to maintain adequate hygienic and sanitary conditions within their homes, under penalty of administrative fines of up to €500 (Municipality of Bologna, 2020).

Given the widespread repercussions of this phenomenon, it is clear that its management requires an integrated, consistent, and preventive approach—one that simultaneously safeguards animal welfare, protects vulnerable individuals, and upholds the well-being of the broader community [16,23].

## 3. Psychopathological Framework of Animal Hoarding: Implications for Operational Management

Animal hoarding (AH), although recognized in the DSM-5 as a specific manifestation of Hoarding Disorder (HD), presents distinctive clinical and relational features. Individuals who hoard animals frequently accumulate objects as well [16]; however, the core pathological element remains the hoarding of animals, driven by unique emotional dynamics centered on perceived caregiving, rescue missions, or deep attachment bonds that are qualitatively different from those underlying object hoarding [10,13,14]. Direct field observations have highlighted the central role of psychopathology in effectively understanding and managing AH. The condition frequently coexists with other major psychiatric conditions, such as psychosis or neurocognitive disorders [12,24,25].

Operational experience gained in Italian contexts, supported by scientific literature [12,14,24,26,27,28], points to the recurrent presence of four main clinical profiles. While not always formally diagnosable during urgent interventions, these patterns are crucial for guiding field-based management strategies:

Obsessive–Compulsive Disorder (OCD)

In these cases, hoarding presents as an egodystonic compulsion—that is, perceived as inconsistent with the individual’s core values—driven by intrusive thoughts of rescue or protection. Awareness of the animals’ suffering and personal distress typically makes these individuals more open to collaboration [12,28]. Often associated with the “overwhelmed caregiver” profile, they recognize their own limitations in managing the situation, albeit with significant feelings of guilt.

Obsessive-Compulsive Personality Disorder (OCPD)

Marked by rigid thinking, perfectionism, and excessive rationalization, this condition leads to egosyntonic behaviors (perceived as consistent with the individual’s identity). These individuals, often identified as “rescuers,” interpret hoarding as an ethical mission and resist any external intervention perceived as a threat [29]. Effective engagement requires a gradual construction of trust and rapport.

Psychosis

In cases involving psychosis, hoarding behavior is driven by structured delusions (e.g., beliefs of spiritual connection with animals), with a marked impairment of reality testing [24]. Intervention in such contexts requires the involvement of mental health professionals to address delusional content, often associated with the profiles of the “exploiter” or the delusional “rescuer”.

Schizophrenia

This condition represents one of the most severe clinical associations with AH. Hoarding behavior is disorganized, impulsive, and devoid of conscious purpose, accompanied by widespread impairment in managing both the environment and the animals. The home environment is often found in extreme conditions, incompatible with human and animal life. Individuals typically exhibit the profile of a disorganized “exploiter” and require structured, and sometimes coercive, interventions along with long-term psychiatric care [27,30].

In addition to this clinical classification, the conceptual model proposed herein introduces a behavioral typology of hoarders, designed to guide operational strategies and support the development of a shared professional vocabulary. The profiles described in Table 1—derived from the integration of bibliographic sources [13,31,32] and field observations—offer a pragmatic framework for interpreting relational patterns with animals and tailoring intervention approaches accordingly. While the behavioral characteristics and levels of awareness are well documented in the literature, the operational suggestions outlined in Table 1 reflect a synthesis of clinical experience and multidisciplinary field practice.

## 4. Materials and Methods

### 4.1. Objectives and Methodological Rationale

This study aims to critically assess the applicability and limitations of the modified observational tool derived from the Case Report Form developed by HARC [5], as used for data collection in cases of animal hoarding within the Italian context. The ultimate objective is to determine whether this tool is suitable to support an integrated multidisciplinary response. If not, the findings would justify the development of a new conceptual protocol tailored to the complexity of the phenomenon.

### 4.2. Description of the Tool Used

The observational tool employed in this study originates from the Case Report Form initially developed by Patronek and later adapted by HARC [9], with additional adaptations to meet the operational requirements of the Italian context. It includes approximately 90 items organized into 20 thematic sections. Specifically, the form addresses areas such as the subject’s demographic information and household composition, the condition of the dwelling and the level of environmental degradation, the presence and health status of animals, the methods by which they were acquired, the accumulation of objects and waste leading to spatial impairment, the individual’s declared motivations, relational dynamics, and the outcomes of the intervention.

The response options are structured through yes/no questions, ordinal scales measuring severity (e.g., from “none” to “severe”), and standardized checklists. However, the tool lacks validated psychometric instruments and does not contain sections specifically designed for relational or psychopathological assessment.

### 4.3. Data Collection Methods

The adapted Case Report Form was applied to 48 documented cases managed between 2019 and 2024 by the Local Health Units ASL Roma 1 and ASL Roma 4, following reports of hygiene violations, suspected animal mistreatment, or environmental degradation. The sources reviewed included veterinary clinical records, official inspection reports, internal ASL documentation, and reports from law enforcement and social services.

These cases were selected because they fulfilled the diagnostic and behavioral criteria for animal hoarding, as defined in the DSM-5, and were confirmed through multidisciplinary assessment involving veterinary, social, and law enforcement professionals. Data collection was conducted by public veterinary officers and healthcare professionals during or after the intervention. Inclusion criteria were based on the definition of an animal hoarder provided by Patronek [9]: a person who has accumulated a large number of animals and is unable to provide them with the minimum standards of nutrition, hygiene, and veterinary care; who fails to act despite the animals’ progressive deterioration (including illness, malnutrition or death) and the degradation of the surrounding environment (severe overcrowding and unsanitary conditions); and who does not recognize the negative consequences of their behavior on their own health or on the well-being of other household members. The data were analyzed using a qualitative synthesis approach aimed at identifying recurrent clinical, relational, and environmental patterns.

### 4.4. Data Analysis

The data, originally collected using paper forms, were transcribed into an electronic spreadsheet (Excel) and analyzed in aggregate form, in full compliance with the European General Data Protection Regulation (GDPR, Regulation EU 2016/679), ensuring that no identifying information was included. A descriptive analysis of the data was carried out by calculating the completion rate for each thematic area. Sections containing partial or incomplete information were classified as critical. No inferential statistical analyses were conducted, as the study focused on assessing the suitability of the tool for the Italian context, with exploratory, prospective, and analytical objectives.

## 5. Results

The field application of the observational tool adapted from the Case Report Form revealed a range of heterogeneous outcomes in terms of operational effectiveness and informational completeness. Overall, the forms were partially—but still meaningfully—completed in approximately 70% of the cases, showing good performance in capturing structural data such as the number of animals present, the hygienic and sanitary conditions of the environment, and the general state of housing degradation.

However, core dimensions essential for a multidimensional care approach—such as the underlying motivation for accumulation, the emotional attachment to the animals, and the individual’s awareness of the situation—were often found to be incomplete, overly generic, or not feasible to assess.

In several cases, for instance, the question regarding “stated motivations” elicited stereotyped responses (e.g., “I’m saving them”, “I can’t abandon them”), lacking clinical depth and difficult to categorize meaningfully. Other sections—such as those addressing family dynamics or previous attempts at intervention—were often incomplete or insufficiently detailed, thus compromising their interpretability.

The veterinary component, although formally included, was generally limited to broad assessments (e.g., “undernourished animals”, “presence of disease”) without the use of validated clinical scales or standardized criteria for evaluating and reporting animal welfare. This approach made it impossible to consistently compare different cases and hindered the integration of data concerning animal health, environmental conditions, and the relational functioning of the individual.

Moreover, the generic nature of the available assessments does not support reliable use in legal contexts, nor does it provide an adequate basis for long-term monitoring or the planning of structured follow-up interventions.

In 21 out of 48 cases (43.7%), voluntary follow-up interviews were conducted; however, the information gathered was not systematically integrated into the tool, resulting in discontinuous observation over time.

The estimated completeness and main critical issues identified across the assessed domains are summarized in Table 2.

### 5.1. Operational Limitations Encountered

Health and social care professionals reported a series of recurring challenges. First, ambiguities or overlaps among items made it difficult to maintain consistency during completion. Second, there was a lack of shared criteria for interpreting and using the data across professionals from different backgrounds, such as veterinarians, psychologists, and social workers. Third, the form proved inadequate as a decision-making tool, as it lacked indicators to assess urgency, risk, priority, or the effectiveness of interventions.

While the tool served as a useful initial descriptive framework, it presented several structural and functional limitations that constrain its use as an integrated operational support in the practical management of cases.

### 5.2. Implications and Rationale for the New Protocol

These observations, which emerged consistently from the analysis of 48 real-life cases, led to the development of a new integrated conceptual model that better reflects the complexity of the phenomenon and addresses three main needs: first, a relational and psychological understanding of the individual engaging in animal hoarding, achieved through a clinical-relational interview; second, an integrated multidimensional assessment encompassing the living environment, physical health, social network, and animal welfare; and third, longitudinal monitoring, with the possibility of updating and comparing cases over time.

The proposed protocol is designed to be dynamic, interdisciplinary, and replicable. It aims to guide initial assessment, intervention planning, and long-term monitoring. A simulated case illustrating its practical application is included in the Supplementary Materials (Files S4–S6) as an example of operational implementation.

## 6. Discussion

The retrospective analysis of a modified observational form—derived from the Case Report Form developed by HARC [5]—applied to 48 real-life cases managed by two local health authorities (ASLs) in the Lazio region between 2019 and 2024, revealed that the tool only partially supports a multidisciplinary and integrated approach to animal hoarding (AH).

While the form proved effective in collecting basic structural data—such as the number of animals and the hygienic-sanitary conditions of the environment, which are essential for immediate intervention—it was less efficient in addressing key dimensions required for a holistic understanding and long-term management of the phenomenon. Specifically, the form lacks a systematic approach to important aspects such as the subjective motivations behind accumulation, the individual’s level of awareness, family and relational dynamics, the nature of the emotional bonds with animals, and the assessment of relapse risk. These dimensions were either absent or addressed in a generic and unstructured manner.

In our sample, psychological and relational areas were particularly underrepresented, with an average completion rate of less than 20%. Similarly, although veterinary data were present, they lacked standardization and clinical detail, making it difficult to evaluate each case individually or compare across cases. This also hindered full integration with environmental or relational analyses. These findings are consistent with operational challenges reported in other studies, which emphasize the need for more robust tools to address the complexity of AH [7,33,34,35].

Another critical issue was the absence of dedicated follow-up sections, which made it impossible to document case evolution or detect early signs of relapse. This discontinuity in data collection compromised the ability to build a cumulative and longitudinal framework, essential for guiding personalized interventions and assessing their long-term effectiveness [8,16].

In practice, the professionals involved encountered difficulties using the form as an operational support tool, citing ambiguities in the items, interpretative complexity, and a general lack of coordination among the different professional roles. This often resulted in fragmented and reactive responses, rather than proactive and coordinated case management.

In this context, the aim of the present study was not to analyze in detail the specific features of the 48 cases, but rather to critically assess the tool through the lens of practical experience in managing them. The consistency and recurrence of the issues encountered underscored the need for a new observational protocol capable of integrating environmental, health, psychological, relational, and follow-up dimensions in an organized and comparable manner. The evidence presented here highlights the critical need for operational tools to support the management of animal hoarding—not only to ensure animal welfare, but also to protect public health and support vulnerable individuals. While animal hoarding shares roots with broader failures in mental healthcare, it differs fundamentally from other untreated psychiatric conditions because its impact extends far beyond the affected individual, creating immediate risks for animals, neighbors, and community health. Unlike depression, anxiety, or object hoarding, animal hoarding cannot be addressed through clinical care alone—it requires coordinated intervention across veterinary, social, legal, and public health domains [10,17,35].

The protocol proposed in this study is designed to operationalize this cross-sectoral approach, providing a conceptual, structured, and replicable tool to guide data collection, risk assessment, and multidisciplinary intervention planning in complex AH cases. However, it will be essential to subject the protocol to future experimental validations, including reproducibility testing, inter-rater reliability, and predictive value with respect to relapse risk. Only through these steps can it achieve full clinical and decision-making validity.

While the present study focuses on cases from a geographically limited area (ASL Roma 1 and ASL Roma 4), the clinical and behavioral patterns observed are consistent with findings reported in the international literature across diverse geographical contexts, including Brazil [7], Portugal [32], England [36], and other countries [35]. Available evidence suggests that animal hoarding occurs with similar prevalence (2–6%) across the United States and Europe [35], and that the core psychopathological and relational dynamics remain relatively stable across different geographical and cultural contexts, with variation primarily occurring in the types of animals accumulated rather than in the fundamental characteristics of the disorder [16,35]. However, further systematic research is needed to explore potential differences in animal hoarding manifestation between Italian regions and between urban and rural settings.

## 7. The Multidisciplinary Operational Protocol for Animal Hoarding: Structure and Application

### 7.1. Conceptual Framework and Aims

Following the identification of key limitations in the use of current operational tools within the Italian context—and informed by extensive practical experience in managing animal hoarding (AH) cases—a new integrated protocol was developed. AH is a condition involving intertwined health, psychological, and social factors, extending far beyond hygienic concerns and often rooted in complex psychosocial suffering and relational disruption [17,36,37]. Therefore, any effective approach must balance medical, psychosocial, and legal priorities, while maintaining a strong focus on the care and dignity of both individuals and animals.

Although this protocol represents a preliminary, conceptual tool not yet validated in the field, it has been designed to provide a structured foundation for future experimental applications. It offers a shared and adaptable framework for intervention, applicable across different territorial contexts.

### 7.2. Tools of the Protocol

The proposed protocol is composed of three integrated tools designed to facilitate collaboration among the various professionals involved, promote a truly multidisciplinary approach, and ensure a continuous and structured care process based on clinical criteria. These tools are:

Preliminary Observational Form (POF)

A rapid screening instrument is to be completed during the initial visit by healthcare professionals, social workers, or law enforcement officers. It allows for the collection of preliminary information on the living environment, number and species of animals, hygienic conditions, and the individual’s openness to dialog. Its observational structure provides an objective basis for deciding whether to initiate a more in-depth assessment process. Estimated completion time: 10–15 min.

Clinical-Relational Interview for Animal Hoarding (ICRAH) (The acronym ICRAH derives from the original Italian name: “Intervista Clinico-Relazionale per l’Animal Hoarding”).

This tool explores the individual’s psychological and relational functioning, perception of the situation, emotional attachment to animals, past experiences, and readiness for change. It is organized into thematic sections with open-ended questions and scoring criteria ranging from 1 to 3 for each domain assessed (e.g., awareness, risk, cooperation). It can be administered in different phases by psychologists, or by social workers and veterinarians with specific relational training. Average administration time: 30–40 min.

Veterinary Health Record for Animals in Hoarding Contexts (VHR—AH)

A clinical tool completed exclusively by veterinarians, aimed at the standardized assessment of the physical, behavioral, health, and housing conditions of the animals present. It includes specific indicators (e.g., nutritional status, presence of lesions) based on the AWIN (Animal Welfare Indicators) approach and provides official documentation suitable for technical, health-related, and legal purposes. Estimated completion time is 5–10 min per representative animal.

All tools are designed to be used in a modular fashion, depending on the severity of the case, the availability of the professionals involved, and the composition of the interdisciplinary team. The forms are available in the Supplementary Materials (Files S1–S3).

These three tools, which differ in scope, duration, and professional requirements, are designed to be applied flexibly depending on the context. Their main features are summarized in Table 3.

### 7.3. Operational Phases of the Protocol

The protocol is structured into four sequential phases, guiding the multidisciplinary team through the entire intervention process:

**Reporting**

The process begins with the collection of preliminary information from formal sources (local health authorities, law enforcement, social services) or informal channels. It is essential to assess the reliability of the source, the presence of critical elements (e.g., odors, noise, visible animals), and the urgency of the intervention.

**On-Site Inspection**

Carried out by public service veterinarians, often accompanied by law enforcement officers to ensure safety and legal access to the premises. During the inspection, an initial clinical evaluation of the animals is conducted, alongside an environmental and hygienic assessment of the living space. If particularly critical conditions are identified—such as extreme degradation, risks to minors or vulnerable individuals, or signs suggestive of psychiatric disorders—the multidisciplinary team is immediately activated. At this stage, the Preliminary Observational Form (POF) is used to provide an initial and concise assessment of the severity of the situation.

**Case Management**

Based on the information collected and the preliminary assessment, the team develops a personalized intervention plan. This may include psychological support, education on responsible ownership, reproductive control, and health and environmental interventions. The approach is tailored to the psychological profile of the individual, available resources, and the condition of the animals and environment. The ICRAH—Clinical-Relational Interview for Animal Hoarding—is fully applied during this phase. It may be administered over multiple sessions by professionals with relational training and contributes to building a shared understanding that informs the intervention plan. At the same time, the Veterinary Health Record (VHR) is completed to document not only the health status of the animals—focusing on widespread pathological conditions, malnutrition, behavioral issues, and uncontrolled reproduction—but also the condition of the surrounding environment.

**Monitoring**

This phase is essential for preventing relapse, which according to the literature occurs at rates approaching 100% [8,35,38]. Monitoring includes scheduled follow-up visits, updates to the previously completed forms, and additional evaluations if necessary. The POF, ICRAH, and VHR are revisited at later stages to document the evolution of the case and the effectiveness of the implemented measures. To ensure long-term effectiveness, the establishment of a specialized service with the stable presence of various professionals (veterinarians, psychotherapists, psychiatrists, social workers) is strongly recommended.

**Figure 1:**
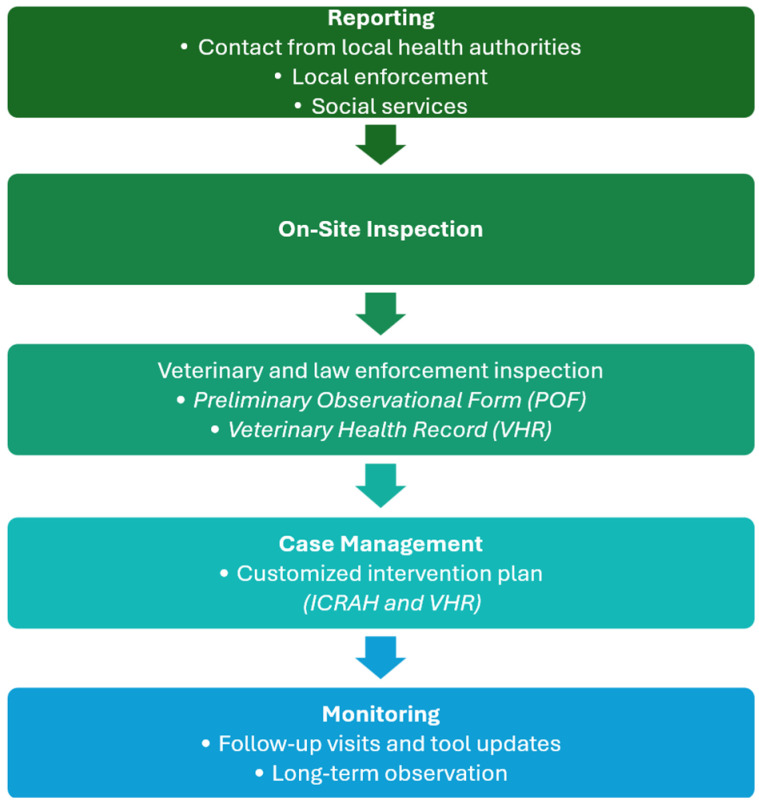
Operational flowchart of the multidisciplinary protocol. Visual representation of the four sequential phases of the proposed protocol for animal hoarding cases. The diagram outlines the key activities, professional roles, and operational tools associated with each phase: reporting, on-site inspection, case management, and monitoring.

A visual summary of the four phases, including main activities, professional roles, and related tools, is presented in Figure 1.

### 7.4. Roles and Approaches of the Multidisciplinary Team

The effective management of animal hoarding (AH) cases relies on stable and coordinated collaboration among professionals from multiple disciplines. Each team member contributes a unique perspective and set of skills essential to managing the clinical, social, legal, and environmental complexities involved [38].

Veterinarians are often the first to interact with the hoarder. In addition to conducting clinical assessments of animal health and welfare, they frequently detect signs of neglect—intentional or not—and play a critical role in building trust with the individual. They also serve as institutional mediators, facilitating coordination among stakeholders [39,40].

Psychiatrists and psychotherapists are responsible for identifying potential comorbid mental health disorders and establishing appropriate therapeutic pathways [34,41].

Social Workers focus on restoring habitable living conditions, supporting everyday functioning, and activating local welfare and care networks.

Law Enforcement Officers ensure safe, lawful access to the premises and support intervention logistics.

Public Health Services assess environmental health risks and contribute to the management of hygiene and sanitation issues [16].

Once the team is formed, the intervention strategies must be adapted to the specific psychological and relational profiles of the individual. As described in Section 3, the approach varies depending on the subject’s level of awareness, emotional attachment to animals, and any underlying psychiatric conditions. For instance, “caregivers” tend to respond positively to structured support, while individuals with psychotic disorders may strongly resist any form of intervention.

Table 4 summarizes the operational strategies associated with four recurring psychopathological profiles observed in the field. These are not formal diagnoses but practical behavioral typologies intended to support real-world decision-making.

These recommendations underscore the need for flexible, evidence-based, and compassionate approaches, tailored to the vulnerabilities and potential for recovery of each individual.

### 7.5. Application Perspectives and Future Validation

The proposed protocol is part of an ongoing exploratory project. Its effectiveness, sustainability, and replicability will need to be assessed through future pilot studies and subsequent systematic validation across different territorial contexts. Although the tool has not yet undergone inter-rater reliability testing or field validation, it has been designed to be usable by professionals with diverse backgrounds—including public veterinarians, clinical psychologists, and social workers—thanks to a shared coding system and complementary sections.

To support its practical application, a simulated case is included in the Supplementary Materials, illustrating the implementation of the protocol in a realistic scenario. The overarching aim is for this model to contribute to the development of regional observatories and inter-institutional networks for the coordinated prevention and management of animal hoarding cases, including in the context of international cooperation.

## 8. Implications for Implementation and Operational Training

The full effectiveness of the proposed protocol depends on its proper application by qualified and adequately trained professionals. For this reason, the activation of well-defined training programs is strongly recommended, aimed at providing the minimum competencies required for the conscious and interprofessional use of the proposed tools.

The recommended training pathway includes three main components. The first focuses on the clinical and regulatory framework of animal hoarding, offering a four-hour overview of diagnostic criteria, veterinary and social implications, and relevant legislation. The second involves a four-hour guided session dedicated to the completion of the protocol, providing practical instructions for using the Preliminary Observational Form, the Clinical-Relational Interview, and the Veterinary Health Record. The third component consists of case simulations and joint exercises, lasting approximately four hours, in which participants apply the protocol to real or simulated scenarios, engage in multidisciplinary discussion of the evaluations, and reflect on potential assessment biases.

A total duration of at least 12 h is recommended. These sessions may be organized either online or in person, and should involve professionals from various disciplines, including veterinarians, psychologists, social workers, and public health authorities, to promote a shared and coordinated approach.

## 9. Conclusions

Animal hoarding constitutes a complex and multifaceted phenomenon that demands operational responses grounded in a multidimensional understanding—integrating clinical, relational, environmental, and legal dimensions. Findings from the retrospective analysis confirm the urgent need for innovative tools to support effective, coordinated, and lasting case management.

The proposed protocol represents an initial step toward standardizing interventions through shared, replicable, and adaptable instruments. The three integrated tools—the Preliminary Observational Form (POF), the Clinical-Relational Interview for Animal Hoarding (ICRAH), and the Veterinary Health Record (VHR)—form a structured operational framework enabling systematic data collection, tailored intervention planning, and longitudinal monitoring of each case.

Field experience highlights that only a stable, interdisciplinary, and well-coordinated approach—bringing together public veterinarians, psychiatrists, psychotherapists, social workers, health authorities, and law enforcement—can ensure effective outcomes. Within this framework, the public veterinarian plays a pivotal role, not only in the clinical evaluation of animals but also as a mediator among institutional stakeholders.

Given the clinical complexity, the high risk of relapse, and the significant public health implications, the establishment of dedicated services and regional observatories is both urgent and strategic. These structures should guarantee continuity of care, professional specialization, and systematic data integration across sectors.

In this light, animal hoarding emerges as a crucial test case for the real-world application of the One Welfare approach [42], which recognizes the systemic interdependence of animal welfare, human mental health, and environmental conditions. Intervening in such cases is not merely a matter of health protection or legal enforcement, but an opportunity to activate relational ecosystems of care—where the well-being of individuals, animals, and communities are addressed as inseparable dimensions of shared vulnerability. The protocol presented here offers a concrete, scalable tool for translating this vision into operational practice, promoting not only the containment of critical situations but a cultural shift in the management of complex vulnerabilities, and providing a conceptual foundation for future validation and adaptation in different national and international contexts.

## Figures and Tables

**Table 1:** Behavioral profiles in animal hoarding: operational implications. Behavioral profiles of animal hoarders, adapted from Patronek et al. [13], Reinisch [6], and the Frost et al. [31]. All descriptions have been reformulated by the authors and integrated with an additional column (*Operational Recommendations*) based on field experience. The terminology of behavioral profiles follows standard usage in the scientific literature on animal hoarding.

Profile	Key Characteristics	Relationship with Animals	Awareness	Operational Recommendations
**Overwhelmed caregiver**	Begins with good intentions but loses control due to personal vulnerability and lack of management skills	Affective, compensatory; feelings of guilt for failing to care properly	Moderate	Establish a non-judgmental, supportive relationship; activate social and psychological support services; encourage gradual reorganization of care routines.
**Rescuer**	Rigid thinking and moral idealization of hoarding; strong resistance to help	Mission-driven, anthropomorphizing	Low	Avoid direct confrontation; build trust progressively; use motivational interviewing to introduce realistic care alternatives without threatening autonomy.
**Exploiter**	Hoards for personal gain, manipulation, or control; often hostile or evasive	Instrumental; animals as status objects or resources	Absent	Coordinate legal and sanitary actions; assess risks to operator safety; involve law enforcement and consider immediate seizure and environmental remediation.
**Incipient hoarder**	Early-stage hoarding; basic standards still met but showing signs of decline	Initial affection and concern; growing neglect	Variable	Prioritize early intervention and monitoring; provide educational resources; involve veterinarians and social services to prevent chronic progression.

**Table 2:** Assessment of key dimensions based on the adapted Case Report Form: estimated completeness and critical issues. Summary of the estimated completeness and main critical issues observed in each area assessed by the adapted Case Report Form. Percentages are approximate and based on the qualitative analysis of completed forms (*n* = 48).

Assessed Area	Estimated Average Completeness	Critical Issues/Observations
**Personal and housing data**	High (~90%)	Data largely available and systematically collected, but little room for socio-economic or family context.
**Number and species of animals**	High (~85%)	Accurate count, but lacking species-specific or behavioral details.
**Animal health conditions**	Moderate (~60%)	Clinical evaluations are generic and not standardized.
**Legal outcomes**	Low (~30%)	Often missing or outdated information.
**Motivational and psychological aspects**	Very low (~15%)	Vague responses; lack of tools for exploration.
**Social network and relational dynamics**	Nearly absent (~10%)	Area not included in the form.
**Follow-up and monitoring**	Absent (0%)	No dedicated section; assessments are discontinuous over time.

**Table 3:** Protocol Toolset Overview. Summary of the three integrated tools included in the proposed operational protocol for animal hoarding cases, detailing their purpose, target professionals, and estimated completion times. Tools are intended to be applied in a modular and flexible manner depending on case severity and team composition.

Tool	Purpose	Operator(s)	Estimated Time
**Preliminary Observational Form (POF)**	Rapid screening of environment, animals, hygiene, and openness to dialog	Healthcare, social services, law enforcement	10–15 min
**Clinical-Relational Interview (ICRAH)**	In-depth assessment of psychological/relational functioning, attachment, awareness, and risk	Psychologists; optionally social workers or veterinarians with relational training	30–40 min (est.)
**Veterinary Health Record (VHR)**	Standardized clinical and welfare evaluation of animals and environmental conditions	Veterinarians	5–10 min per representative animal

**Table 4:** Psychopathological profiles and corresponding operational strategies. These profiles do not constitute formal psychiatric diagnoses but are field-derived categories observed by professionals in multidisciplinary interventions.

Observed Profile	Operational Recommendations
Obsessive–Compulsive Disorder (OCD)	Set clear boundaries and foster an alliance with the veterinarian. CBT as first-line therapy; pharmacological treatment when indicated.
Obsessive-Compulsive Personality Disorder (OCPD)	Maintain a structured but non-coercive approach. Psychotherapy should focus on enhancing cognitive flexibility and managing control needs.
Psychosis	Ensure continuous monitoring and address delusional beliefs. Coercive measures may be required to safeguard health and safety.
Schizophrenia	Long-term care involving psychiatric and social support, pharmacological treatment, and psychosocial rehabilitation.

## Data Availability

The data presented in this study are not publicly available due to the sensitive nature of the information, which involves both people and animals, but may be provided by the corresponding author upon reasonable request.

## Supplementary Materials

The following supporting information can be downloaded at: https://www.mdpi.com/article/10.3390/ani15213222/s1: File S1. Preliminary Observational Form (POF); File S2. Structured Clinical-Relational Interview for Animal Hoarding (ICRAH); File S3. Veterinary Health Record for Animals in Hoarding Contexts (VHR-AH); File S4. Simulated case—POF (completed example); File S5. Simulated case—ICRAH (completed example); File S6. Simulated case—VHR (completed example).
