# Supplementary material for: Development of an Operational Protocol for Animal Hoarding: A Conceptual Proposal Based on Multidisciplinary Field Experience
Source: Animals (Basel). 2025 Nov 6;15(21):3222. doi: 10.3390/ani15213222 (PMC12610984; doi:10.3390/ani15213222)
Supplement: Supplementary file 1 [file animals-15-03222-s001.zip › S1-PRELIMINARY OBSERVATIONAL FORM POF.pdf]

PRELIMINARY OBSERVATIONAL FORM – Animal Hoarding Context

Operational Version for Public Veterinarians and Staff from Other Agencies

Estimated completion time: 10–15 minutes

Date: ____________________ **Operator:** __________________**Agency:** _________________
Inspection address: ____

1. Type of Premises and Housing Conditions

☐ Private home ☐ Apartment ☐ Farm structure ☐ Other: __________________
Housing accessibility:
☐ Easily accessible ☐ Difficult access ☐ Unsafe / hazardous

2. Resident's name (if available) ____

Approximate age __________________

Access allowed? ☐ Yes ☐ Partial ☐ No

Cooperation level ☐ Friendly ☐ Neutral ☐ Evasive ☐ Hostile

3.Animals Present ***(visual estimate or as declared)***

Please fill in even in case of approximate estimates.

**Species**	**Estimated No.**	**Puppies/Kittens?**	**Active reproduction?**	**Visible signs of distress?**
**Dogs**		☐ **Yes** ☐ **No**	☐ **Yes** ☐ **No**	☐ **Yes** ☐ **No**
**Cats**		☐ **Yes** ☐ **No**	☐ **Yes** ☐ **No**	☐ **Yes** ☐ **No**
**Other (specify):**		☐ **Yes** ☐ **No**	☐ **Yes** ☐ **No**	☐ **Yes** ☐ **No**

Notes: ……………………………………………………………………………………

4. Fragile Individuals in the Household

☐ None
☐ Minors → Age: ______ Number: ______ Involvement: ▫ direct ▫ indirect
☐ Elderly individuals (>75 years) → Number: ______
☐ Persons with disabilities or mental fragility → Specify: ____________________________

5. Human–Animal Relationship

☐ Affective (*talks to animals, pets them, shows emotional bonding*)
☐ Neglectful (*lacks care, does not provide food or hygiene*)
☐ Ambivalent (*alternating affection and neglect; emotional disorganization*)
☐ Instrumental (*uses animals for external purposes: begging, control, imposed companionship*)
☐ Other: ____

6. Environmental Conditions *(direct observation)*

Guidance for completion:

- *Good = clean environment, no strong odours or visible accumulation.*
- *Fair = localized dirt, noticeable smell, animals mobile, environment accessible.*
- *Severe = heavy presence of feces, stagnant urine, organic decay, infestations, or clear biological risk.*

**Indicator**	**Observed level**	**Notes**
Strong odour of feces/urine	☐ Yes ☐ No ☐ Not assessable	
Visible presence of feces	☐ Yes ☐ No ☐ Not assessable	
Object accumulation / clutter	☐ None ☐ Moderate ☐ Severe	
Fire/fall hazard	☐ Present ☐ Absent	
Ventilation / natural light	☐ Adequate ☐ Inadequate	
Signs of infestation (insects/rodents)	☐ Yes ☐ No ☐ Not assessable	

7. Additional Observations (Optional)

☐ Hoarding of objects or waste
☐ Unsafe structures or electrical systems

☐ Presence of carcasses or animal remains

☐ Rodents or pests

☐ Other: ____

8. Subject's Behavioral Indications ***(observation + brief dialogue)***

Behavioral cues from the subject

(gathered through direct observation and a short conversation)

Brief open-ended questions *(if feasible)*

(Record short, colloquial responses. Use the person's exact words if meaningful)

- "How do you feel about your current situation with the animals?"
  …………………………………………………………………
- "What is the hardest thing to manage right now?"
  …………………………………………………………………
- "If you could change something, what would it be?"
  …………………………………………………………………
- "Have you ever received help for this situation?"
  …………………………………………………………………

Problem awareness

☐ Aware

☐ Minimizes

☐ Denies

☐ Not assessable

Collaboration during the interview

☐ Friendly

☐ Formal

☐ Avoidant

☐ Hostile

Operator's behavioral notes *(tone, emotional expression, contradictions)*:

………………………………………………………………………………………………

9. Preliminary Risk Assessment

Overall Risk Level	Brief Description

☐ 1 – No risk	Normal management, clean environment, animals in good condition
☐ 2 – Mild risk	Some issues, but overall manageable situation
☐ 3 – Moderate risk	Hoarding behavior ongoing, borderline environmental conditions
☐ 4 – High risk	Severe disorder, questionable animal conditions, evasive attitude
☐ 5 – Critical risk	Serious unsanitary conditions, maltreatment, current health hazard

Justification for the assigned score (brief):
…………………………………………………………………………………………

10. Recommendations and Referrals

Suggested actions (check all applicable):
☐ Periodic follow-up (within 30 days)
☐ Multidisciplinary team activation
☐ Psychosocial evaluation (social/mental health services)
☐ Comprehensive veterinary assessment (→ Veterinary Health Form)
☐ Emergency action / Judicial authority notification

Additional notes:
………………………………………………………………………………………………
………………………………………………………………………………………………
Note: Proposed actions should be evaluated considering the severity of the risk, the presence of vulnerable individuals, and the resident's cooperation.

Operator's Full Name: ____________________________
Affiliated Service/Agency: ____________________________
Date: ____________ **Signature:** ____________________________
