# Supplementary material for: Development of an Operational Protocol for Animal Hoarding: A Conceptual Proposal Based on Multidisciplinary Field Experience
Source: Animals (Basel). 2025 Nov 6;15(21):3222. doi: 10.3390/ani15213222 (PMC12610984; doi:10.3390/ani15213222)
Supplement: Supplementary file 1 [file animals-15-03222-s001.zip › S2-STRUCTURED CLINICAL-RELATIONAL INTERVIEW FOR CASES OF ANIMAL HOARDING (ICRAH).pdf]

STRUCTURED CLINICAL-RELATIONAL INTERVIEW FOR CASES OF ANIMAL HOARDING (ICRAH)

Estimated completion time: 30–40 minutes

Introduction for the practitioner

This structured yet flexible clinical-relational interview is designed to support professionals—such as psychologists, social workers, veterinarians, educators, and other practitioners—in gaining an in-depth understanding of individuals living with a large number of animals, with respect for their personal history, emotional experience, and inner resources.

The goal is not to formulate a diagnosis or make judgments, but to gather key elements to guide the assessment of needs, vulnerabilities, and potential pathways for support and care.

The interview is organized into sections that explore personal, relational, environmental, and motivational aspects, using open-ended questions, clinical observations, and qualitative checklists.

It is not intended to produce clinical diagnoses, but rather to inform a multidimensional evaluation of needs and strengths and to support the planning of tailored interventions. Coding and observations are tools to guide decision-making—not to classify the individual.

Guiding Principles for Administration

- Listen without judgment. Each individual has a unique story, often shaped by complex experiences.
- Encourage spontaneous narration. This interview is not an interrogation, but a space for attentive listening.
- Observe with care and respect. Environmental details, emotional reactions, and language used are valuable sources of information.
- Take detailed notes. Written observations will help reconstruct a complex picture, also valuable in multidisciplinary contexts.
- Adapt the format to the context. Some individuals may prefer to interact only with specific professionals (e.g., veterinarians); in such cases, use the interview as a flexible guide to facilitate communication.

Data Usage

Collected data should be recorded in the individual's personal file, in compliance with privacy regulations and with the aim of providing psychosocial, clinical, and environmental support.

If the data are to be used for research or training purposes, informed consent and full anonymization are required.

SECTION 1 – PERSONAL DATA AND SOCIAL RELATIONSHIPS

The collection of personal data must be conducted respectfully and in proportion to the context. In cases of significant discomfort or refusal, the interview may proceed without gathering the full set of information.

1.1 Identity and Contact Information
"How would you prefer to be addressed?"
"Is there anything you would like to share to help us get to know you better (e.g., age, identity, preferred pronouns)?"
(Only if necessary): "May we record your name and contact information for possible future follow-up?"

- Full Name: ____
- Address: ____
- Contact details (phone/email): _______________________
- Age: _______
- Gender: □ Male □ Female □ Other □ Prefer not to say
- Additional notes (name use, preferences, etc.): ______________________

"May we see an identity document and record the relevant details?"

- Type of document: _________________________
- Document number/ID: ________________________

1.2 Employment Status and Educational Background
Questions to be asked in an open and welcoming manner:
"What kind of activity are you currently involved in, if any?"
(e.g., employed, retired, seeking work, other)
"If you feel comfortable, could you tell me about your educational background?"
(There are no right or wrong answers—this is simply to better understand your experience and available resources.)

1.3 Economic Condition
The aim is not to obtain an exact financial figure, but rather to understand the person's subjective experience and any vulnerabilities related to everyday life management, particularly in connection with the welfare of the animals.
Question (to be asked with sensitivity):
"I understand this may be a sensitive topic, but if you feel comfortable, could you tell me how you perceive your current financial situation?
Are you able to cover daily expenses, including those related to your own needs and the animals?"
The person may freely add whether they receive pensions, subsidies, family support, or other forms of assistance.
Qualitative Indicators – Signs of Economic Stability or Vulnerability
(Check if applicable)
☐ Sufficient and stable income
☐ Irregular or variable income
☐ Reliance on external aid (family, services, volunteers)
☐ Difficulty covering daily expenses
☐ Difficulty covering veterinary costs

☐ Refusal or discomfort in discussing financial matters
☐ Other: ______________________________

Observational Notes:

Free space for noting emotional tone, signs of distress, resistance or openness, and coping strategies.

1.4 Co-habitation and Family Context

This section aims to assess whether the person's household network serves as a resource or represents a source of vulnerability, and how it affects the management of animals and the individual's overall well-being.

Open-ended questions (to be asked in a welcoming manner):

"Do you currently live with someone?"
"What is your relationship like with the people you live with? And with the animals?"
"Are there people who care for you or whom you care for?"
"Are there individuals in the home with special needs, such as children, elderly, or vulnerable persons?"

Qualitative Indicators – Presence of Co-habitants and Contextual Fragilities

(Check if applicable)

☐ Lives alone
☐ Lives with family / friends / partner
☐ Positive relationships with co-habitants
☐ Family conflict or relational difficulties
☐ Presence of minors
☐ Presence of elderly or disabled individuals
☐ Other relevant elements: ___________________________

Observational Notes:

Record observable relational dynamics, emotional attitudes toward co-habitants, or signs of isolation, protection, or conflict.

1.5 Personal History

This section is intended to explore the individual's subjective experiences that may influence their relationship with animals, offering valuable insights into the emotional and symbolic meaning of these bonds.

Open-ended questions (to be asked in a respectful and supportive manner):

"Are there any moments in your life that you consider particularly important and that might help me better understand your current situation?"
"Have you experienced events that have left a deep mark on you, whether positive or negative?"
"Is there anything in your personal history that you feel the need to share with me?"

Qualitative Indicators – Significant Events or Emerging Vulnerabilities

(Check if applicable)

☐ Presence of traumatic events or recent losses
☐ Isolation, separations, bereavement, or abandonment
☐ Positive experiences of coping or resilience

☐ Complex or unstable relational history
☐ Coherent and reflective narrative
☐ Difficulty discussing personal history / tendency to avoid
☐ Other: ____________________________

Observational Notes:
Record narrative coherence, emotions expressed during storytelling, and any signs of suffering, emotional suppression, or idealization.

1.6 Health and Daily Habits
This section supports the assessment of general health and level of autonomy—both critical elements in determining the person's ability to ensure the well-being of the animals in their care.
Open-ended questions (to be asked with sensitivity and respect for privacy):
"Are there any aspects of your health you'd like to talk about, that you feel are important for understanding your current well-being?"
"Are you currently undergoing treatment, taking prescribed medications, or receiving any kind of healthcare support?"
"Are there habits that you feel contribute positively to your well-being, or conversely, challenges you face in daily life?"

Qualitative Indicators – Relevant Medical or Behavioral Information
(Check if applicable)
☐ Known medical diagnoses
☐ Regular use of prescribed medications
☐ Ongoing treatments or therapies
☐ Substance use or abuse (alcohol, medications, other)
☐ Health conditions that may interfere with daily functioning
☐ No relevant health conditions reported
☐ Other: ____________________________

Observational Notes:
Note any indirect signs of vulnerability (e.g., fatigue, confusion), coherence of the narrative, openness in discussing health, and perceived autonomy.

1.7 Social Network and Relationships
This section aims to assess the individual's level of social integration—whether a protective or supportive network exists, or whether the person is experiencing isolation, which constitutes a key vulnerability factor in cases of animal hoarding.
Open-ended questions (to be asked in a non-judgmental manner):
"Are there people you feel you can rely on, even just to talk to or ask for help if needed?"
"Do you maintain regular contact with family members, friends, or neighbors? Do you enjoy receiving visitors?"
"Do you feel supported by your social network, or do you sometimes feel alone?"

Qualitative Indicators – Presence of Supportive Relationships
(Check if applicable)

☐ Stable presence of family or friendship bonds
☐ Active and perceived supportive social network
☐ Occasional but meaningful relationships
☐ Perceived social isolation
☐ Limited emotional or practical support
☐ Conflicted or interrupted relationships
☐ Other: ____________________________

Observational Notes:

Note emotional tone, signs of relational distress or ambivalence, or protective elements within the personal network (e.g., individuals mentioned with affection, regular contact, etc.).

1.8 Previous Experiences with Authorities or Services

This section provides insight into the individual's past experiences with support systems and helps evaluate their willingness to collaborate, or the presence of distrust, avoidance, or oppositional dynamics—critical aspects when planning an effective intervention.

Open-ended questions (to be asked with sensitivity):

"Has anyone—such as neighbors, social or health services, or authorities—ever pointed out anything to you regarding your animals or your home?"

"How did you experience these situations? Did they seem unfair, or did they give you something to reflect on?"

"Has anyone ever offered help? If so, how did you feel in that moment?"

Qualitative Indicators – Past Experiences and Attitude Toward Services

(Check if applicable)

☐ No prior experiences with services
☐ Previous positive or collaborative experiences
☐ Conflictual or perceived invasive experiences
☐ Collaborative attitude toward potential interventions
☐ Distrustful or closed attitude toward services
☐ Other: ____________________________

Observational Notes:

Note the interviewee's attitude toward the interviewer, emotional tone of the account (e.g., anger, disappointment, resignation, openness), and how past experiences with services are recalled.

1.9 Recent Critical Events

Open-ended question:

"In the past few months, have there been any difficult events or situations that have changed something in your daily life or in your relationship with your animals? (For example: a loss, a conflict, a complaint, health problems, or other?)"

Checklist – Signs of Risk or Well-being

☐ No relevant events reported
☐ Isolated but managed events (e.g., processed bereavement)

☐ Serious or repeated events with clear impact on well-being and daily management
☐ Other: ___________________________
Observational Notes:
Note any emotional responses, physical reactions, difficulty narrating, or overlap between personal life and animal care.

1.10 Relationships with Neighbors
Open-ended question:
"What is your relationship like with your neighbors, particularly regarding the presence of animals? Have there ever been comments, complaints, or unpleasant situations?"
Checklist – Signs of Risk or Well-being
☐ Cooperative and peaceful relationship with neighbors
☐ Fragile tolerance or occasional complaints
☐ Ongoing conflicts, reports, repeated tensions
☐ Other: ___________________________
Observational Notes:
Observe for signs of anger, shame, sense of injustice, or tendencies toward social withdrawal.

SECTION 2 – ANIMALS AND MANAGEMENT

2.1 Current Presence of Animals
Open-ended questions:
"How many animals are currently living with you?"
"What kind are they (dogs, cats, others)? How would you describe them (age, origin, particular traits)?"
"How do you organize their daily care?"
Checklist – Animal Presence and Management Capacity
☐ Number of animals proportionate to available resources and space
☐ High number of animals, but managed with commitment
☐ Excessive number relative to caregiving capacity
☐ Presence of species that are incompatible with each other
☐ Difficulty providing a complete or updated description
Observational Notes:
Note descriptive ability, awareness of individual animal conditions, and signs of overload or confusion.

2.2 Origin and Arrival of the Animals
Open-ended questions:
"How did these animals come to live with you? Did you seek them out, or did they arrive spontaneously?"
"Do you continue to take in new animals? If so, what motivates you to do so?"

(Encourage spontaneous storytelling. Avoid suggesting responses. Examples are for practitioner reference only: “I found them,” “I couldn’t leave them,” “Someone asked me to help.”)

Checklist – Arrival Modalities and Predominant Motivation

☐ Occasional and purposeful intake (e.g., adoption, rescue)
☐ Frequent, not always planned arrivals
☐ Accumulation due to repeated rescues or abandonment cases
☐ Tendency to assume responsibility without prior evaluation
☐ Lack of awareness regarding the motivations for intake

Observational Notes:

Note any compulsive patterns, difficulty setting limits, emotional content of the narrative, or lack of discernment in selection.

2.3 Daily Management Practices

This section aims to understand not only whether caregiving is occurring, but how the individual experiences it: as part of a conscious routine, as an act of affection, as a burden, or as a compulsive behavior. It is essential to compare reported practices with direct environmental observations.

Open-ended questions:

“How is your day structured with your animals?”
“Are there specific times dedicated to their cleaning, feeding, or medical care?”
“Do you feel able to manage everything, or are there difficulties?”
(Encourage a narrative of daily routines, listening for both practical and emotional aspects. Pay attention to signs of overload.)

Checklist – Observable Indicators of Daily Management

☐ Regular and structured caregiving routine
☐ Care is provided, but with some logistical or physical challenges
☐ Disorganized management, partial or neglected care
☐ Delegation to others or presence of external help
☐ Lack of awareness of the animals’ basic needs

Observational Notes:

Note consistency between what is reported and what is observed, signs of fatigue, available equipment, and organizational capacity.

2.4 Hygiene and Living Environment of the Animals

This section relies heavily on direct observation. It is important to compare what is declared with what is actually seen: the environmental well-being of the animals reflects the caregiver’s ability to meet their needs, beyond mere emotional intention.

Open-ended questions:

“Where do the animals primarily live? Are there specific areas designated for them?”
“How is the cleaning of their living areas organized?”
“Is there anything you would like to improve, or that concerns you in particular?”

(Encourage free description, paying close attention to any signs of discomfort, poor hygiene, or animal isolation.)

Checklist – Observations on the Animals' Living Environment

☐ Clean, well-ventilated spaces with appropriate materials
☐ Functional environments, but showing signs of caregiver fatigue
☐ Overcrowded areas, strong odors, presence of feces/urine
☐ Confined animals, closed or restricted-access spaces
☐ Inappropriate or hazardous areas (e.g., mold, sharp objects, escape risk)

Observational Notes:

Record general hygiene conditions, presence of litter boxes, bedding, clean bowls, and access to light and ventilation. Also observe animal behavior within these spaces.

2.5 Behaviors and Relationships Among Animals

This section offers a relational perspective—not only among the animals, but also between the animals and the person. The presence of dysfunctional or overly symbiotic relationships may indicate emotional vulnerability or difficulties in caregiving and can help determine the most appropriate form of support.

Open-ended questions:

"How do your animals usually behave with one another?"
"Have you ever noticed signs of tension, aggression, or unusual behaviors (such as repetitive movements or social withdrawal)?"
"How would you describe your relationship with them? Do you feel sought out, welcomed, or avoided?"
"Can you tell if there are affectionate bonds among the animals, or if some appear excluded or isolated?"

(Encourage a free-flowing narrative. Avoid prompting interpretations—let the individual's emotional-relational meanings emerge naturally.)

Checklist – Indicators of Intra-/Inter-species Relational Dynamics

☐ Harmonious interactions among animals
☐ Occasional conflicts or tensions
☐ Frequent or marked conflict among specific animals
☐ Balanced relationship between person and animals
☐ Signs of overinvolvement or emotional symbiosis
☐ Atypical behaviors (stereotypies, avoidance, excessive vocalizations)
☐ Presence of animals who are isolated, passive, or withdrawn

Observational Notes:

Freely describe the quality of relationships, any aggressive behaviors, stress signals, or—conversely—signs of bonding, play, or physical closeness. Note also the subject's posture, tone, and emotional expression when speaking about the animals.

2.6 Management of Animal Death

How the individual handles the death of an animal often reflects their capacity for emotional processing, symbolic meaning-making, and separation. Ritualized, avoidant, or

denial-based responses may indicate unresolved grief or dysfunctional attachment and should be explored with respect and without judgment.

Open-ended question:

"When one of your animals dies, what usually happens? How do you cope with that moment? What do you choose to do with the body?"

(Allow for spontaneous narration. Do not interrupt, even if symbolic or highly emotional elements emerge. Document ritualized practices or avoidant responses.)

Checklist – Indicators in the Management of Animal Loss

☐ Proper disposal through veterinary services or legal authorities
☐ Burial at home, in a garden or rural area
☐ Retention of the animal's body in the home (e.g., mummification, freezing, taxidermy, other)
☐ Difficulty or refusal to confront the loss
☐ Ritualized or idealized narrative of death
☐ Intense emotional reaction to the loss (e.g., crisis, social withdrawal, increase in accumulation)
☐ Other (specify): ________________

Observational Notes:

Carefully note the emotional expression, any difficulties processing grief, the presence of symbolic or spiritual explanations, denial of death, or lack of emotional detachment. Observe for signs of frozen grief or pathological attachment.

SECTION 3 – LIVING CONDITIONS AND ENVIRONMENTAL ASPECTS

3.1 General Hygiene Conditions

Questions:

1. How would you describe the environment where you live with your animals? Are there designated areas for them, or do you share all spaces?
2. Have you ever had difficulty keeping the home clean or tidy? Are there times when it becomes particularly hard to manage?
3. Are there rooms or areas in the house that you avoid using or that have become difficult to maintain?
4. Have you had any problems with the structure of the house (e.g., dampness, plumbing, mold, damage)?
5. Are the windows used regularly? Is there adequate air circulation and natural light in the home?
6. Have you ever noticed the presence of pests, such as insects, rodents, or persistent odors?
7. Have you observed any accumulation of objects, decomposing materials, or hard-to-remove waste?

Checklist – Indicators of Environmental Risk or Well-being

(check only if observed)

☐ Organized and accessible living spaces

☐ Clean environment with regular hygiene management
☐ Difficulty maintaining cleanliness or order
☐ Unusable or overcrowded areas
☐ Presence of mold, structural damage, or hazards (e.g., exposed wiring or pipes)
☐ Poor air circulation or dark indoor environments
☐ Strong or persistent odors
☐ Presence of insects, rodents, or other pests
☐ Presence of decomposing organic materials or accumulated waste
☐ Other: ____________________________

Observational Notes:

Document the general condition of the home, visible hygiene and sanitation levels, any environmental risk factors, inaccessible areas, and the individual's subjective perception of cleanliness and order.

SECTION 4 – PSYCHOLOGICAL ASPECTS AND MOTIVATIONS

4.1 Emotional Bond and Perceived Role of Animals

This section explores the subjective, emotional, and symbolic meaning attributed to animals, highlighting both personal strengths and potential signs of vulnerability or dependency. The aim is not to assess the "quality" of the bond, but to understand its function within the person's life context.

Questions:

1. How would you describe the emotional bond you have with your animals?
2. What role do they play in your life? (e.g., companionship, protection, emotional support, sense of purpose...)
3. Do you believe that their presence fulfills a specific personal need?
4. Have you ever felt that your animals understand you better than people do?
5. Have your animals helped you through difficult moments in your life?
6. Have you ever thought about being separated from any of them? What emotions did that provoke?
7. Do you think someone else could take care of them? What are your thoughts on that?

Checklist – Emotional Role and Function of the Animals

☐ Balanced emotional bond, with awareness and openness
☐ Presence of emotional needs compensated by animals, with some degree of reflection
☐ Overwhelming bond, with strong substitutive or regulatory function; significant dependency
☐ Rejection of the idea of separation or delegation
☐ Other: ____

Observational Notes:

Observe emotional tone, ambivalence, resistance to separation, idealization, or overinvestment in animals.

4.2 Social Perception and Awareness of the Problem
Questions:
8. Has anyone ever pointed out that the number of animals you keep might be too high? How did you respond?
9. In your opinion, what are the main challenges involved in managing all these animals?
Checklist – Social Awareness
☐ Full awareness and openness to dialogue
☐ Defensive reactions, but partial acknowledgment
☐ Denial of the problem or refusal to engage in discussion
☐ Other: ____
Observational Notes:
(Free space for clinician's notes.)

4.3 Impulsivity and Capacity for Letting Go
Questions:
10. How do you experience the desire or impulse to take in new animals or objects?
11. Would it be difficult for you to part with some of your animals? In what way?
Checklist – Impulsivity and Attachment
☐ Good capacity for self-control and separation
☐ Moderate discomfort, occasional impulses
☐ Intense distress or inability to separate, uncontrolled intake behavior
☐ Other: ____
Observational Notes:
(Free space for clinician’s notes.)

4.4 Underlying Motivations and General Emotional State
Questions:
12. What has motivated you to take in so many animals? (e.g., protection, companionship, past difficulties...)
13. How would you describe your general emotional state in recent times?
14. Do you feel lonely or isolated? Do you find it difficult to maintain meaningful relationships?
15. Would you consider seeking professional support to improve your situation?
Checklist – Motivations and Openness to Support
☐ Clear motivations, stable emotional state, openness to support
☐ Partial motivations, unstable emotional state, limited openness
☐ Confused or denied motivations, compromised emotional state, unwillingness to engage
☐ Other: ____
Observational Notes:
(Free space for clinician’s notes.)

SECTION 5 – RISK ASSESSMENT AND WILLINGNESS TO COLLABORATE

This section explores the individual's self-awareness, motivation for change, and willingness to engage in collaborative efforts—key factors in evaluating the feasibility and timing of potential interventions. The questions are not intended to pass judgment, but to build an alliance and detect signs of openness or resistance.

5.1 Perception of the Situation and Risk Awareness

Questions:

1. If you had to describe your current situation with your animals, what would you say?
2. Do you feel everything is under control, or are there aspects that are sometimes challenging?
3. Do you think your animals are doing well? And how do you feel?

Checklist – Indicators of Awareness

☐ Realistic perception of the situation
☐ Partial or ambivalent awareness
☐ Denial or underestimation of the issues

Observational Notes:

(Free space for clinician's notes.)

5.2 Willingness to Change and Collaborate

Questions:

4. Have you ever thought that, with a bit of help, some things could improve?
5. What do you think about the possibility of gradually reducing the number of animals?
6. If you have tried to change something in the past, what happened?

Checklist – Indicators of Openness

☐ Willingness to engage and consider habit changes
☐ Ambivalence or uncertainty about change
☐ Rigidity and refusal of any change

Observational Notes:

(Free space for clinician's notes.)

5.3 Previous Experiences and Future Planning

Questions:

7. Have you previously received complaints or had issues with others regarding your animal care? How did you experience those situations?
8. In an ideal scenario, how many animals do you think would be appropriate to have?
9. What would you like to change in order to feel better—both for yourself and for them?

Checklist – Indicators of Future Planning

☐ Integrated past experiences and constructive outlook
☐ Partially processed conflicts or difficulties
☐ Rejection of the past and lack of future perspective

Observational Notes:

(Free space for clinician's notes.)

5.4 Available Resources and Interdisciplinary Collaboration
Questions:
10. What types of support would be most helpful to you right now? (emotional, practical, medical…)
11. In your opinion, who could support you along this path?
12. Would you be willing to collaborate with multiple professionals, such as a veterinarian, psychologist, or social worker?

Checklist – Possible Resources and Alliances
☐ Clearly identified needs, openness to networking
☐ Vague needs, limited willingness
☐ Rejection of external help, unwillingness to collaborate
☐ Existing collaboration with professionals (specify): __________

Observational Notes:
(Free space for clinician's notes.)

SECTION 6 – NARRATIVE SUMMARY AND METAPHOR

Methodological Note
This concluding section offers an empathic and reflective space aimed at capturing the individual's overall lived experience through symbolic narration. The metaphor allows access to an emotional level that may not be expressible through rational responses, while the clinician's summary helps integrate the collected information into a coherent overview, useful for planning further intervention.

Open-ended question for the individual
"If you had to describe your relationship with your animals using an image or a metaphor, what would it be?"
The practitioner should not suggest or interpret the answer but simply receive the person's words as they are.

Clinical-Relational Summary by the Practitioner
(To be written in narrative form, max 10 lines)
Summarize the main elements that emerged during the interview, with particular attention to:

- Explicit or potential strengths
- Critical areas and risk indicators
- Willingness to change
- Human–animal relationship
- Suggested next steps
