# Supplementary material for: Development of an Operational Protocol for Animal Hoarding: A Conceptual Proposal Based on Multidisciplinary Field Experience
Source: Animals (Basel). 2025 Nov 6;15(21):3222. doi: 10.3390/ani15213222 (PMC12610984; doi:10.3390/ani15213222)
Supplement: Supplementary file 1 [file animals-15-03222-s001.zip › S3-VETERINARY HEALTH FORM FOR ANIMALS IN HOARDINGCONTEXTS VHR -AH.pdf]

Veterinary Health Form for Animals in Hoarding Contexts (VHR-AH)

Estimated completion time: approx. 10–15 minutes per animal

Animal Owner____

Section	Details
**1. General Information about the Animal**	
Animal ID	
Species	
Breed	
Estimated Age	
Sex	☐ Male ☐ Female ☐ Not Determinable
Reproductive Status	☐ Intact ☐ Spayed/Neutered ☐ Not Detected
Microchip	☐ Yes (Number: ________) ☐ No ☐ Not Detected
**2. General Physical Conditions**	
Weight	________ kg (☐ measured / ☐ estimated)
Body Condition Score (BCS)	☐ 1 (severely underweight) ☐ 2 (underweight) ☐ 3 (normal) ☐ 4 (overweight) ☐ 5 (obese)
Coat Condition	☐ Clean ☐ Dirty ☐ Matted/Tangled ☐ Alopecia
Skin Conditions	☐ Normal ☐ Lesions ☐ Parasites ☐ Specify: __________
Eye Conditions	☐ Normal ☐ Reddened ☐ Discharge ☐ Type: __________
Ear Conditions	☐ Normal ☐ Dirty ☐ Infections ☐ Specify: __________
**3. General Health Status**	

Signs of Dehydration	☐ Absent ☐ Mild ☐ Moderate ☐ Severe
Signs of Malnutrition	☐ Absent ☐ Present ☐ Specify: __________
Dental/Oral Problems	☐ Absent ☐ Present ☐ Description: __________
Mobility	☐ Normal ☐ Lameness ☐ Muscle Atrophy ☐ Specify: __________
Evident Pain Signs	☐ Absent ☐ Present ☐ Location: __________
Clinical Signs of Disease	☐ Cough ☐ Vomiting ☐ Diarrhea ☐ Other: __________
Zoonotic Risks Identified (e.g., fleas, mange, bites)	☐ No ☐ Yes → Specify: __________________________
**4. Behavioral Status**	
**Guidance for Completion – Animal Behavior**<br>Friendly: Approaches willingly, accepts contact, relaxed.<br>Fearful: Withdraws, avoids contact, shows trembling or submissive posture.<br>Aggressive: Growls, bites, displays defensive/offensive posture, actively attacks.<br>Social (toward other animals): Peaceful interaction, cohabitation without issues.<br>Fearful (toward other animals): Hides, runs away, avoids contact.<br>Stereotypies: Repetitive non-goal-oriented behaviors (e.g., circling, licking, chewing air).	
Behavior Toward Humans	☐ Friendly ☐ Fearful ☐ Aggressive ☐ Other: __________
Behavior Toward Other Animals	☐ Social ☐ Fearful ☐ Aggressive ☐ Other: __________
Stereotypies or Repetitive Behaviors	☐ Absent ☐ Present ☐ Type: __________
**5. Preliminary Diagnosis and Notes**	
Diagnosis	
Recommended Examinations	
Suggested Therapies	
Additional Notes	
**6. Photographic Documentation**	
Photo 1	Full body Date: ________

Photo 2	Lesion detail Date: ________
**7. Welfare Assessment (AWIN Protocol)**	
Access to Food and Water	☐ Adequate ☐ Inadequate
Physical Comfort	☐ Normal ☐ Limited ☐ Other: __________
Overall Health Status	☐ Good ☐ Compromised ☐ Other: __________
Normal Behaviors	☐ Present ☐ Absent ☐ Other: __________
Social Interaction	☐ Adequate ☐ Evident Problems ☐ Other: __________
Response to Stimuli	☐ Normal ☐ Altered ☐ Other: __________
Exploratory Behavior	☐ Present ☐ Absent ☐ Other: __________
Note: *The welfare indicators included in this section are adapted from the AWIN (Animal Welfare Indicators) protocol for assessing the welfare of dogs, developed under the European AWIN project.* *Reference: Barnard, S.; Ferri, N.; Velarde, A.; Batchelor, G.; Gebhardt-Henrich, S.G.; Winckler, C.; Keeling, L.J.; Wemelsfelder, F.; Mendl, M.; Whay, H.R. Development of a Protocol for On-Farm Welfare Assessment of Dogs; AWIN Project: Milan, Italy, 2015.*	
**8. Final Evaluation**	
Overall Welfare Status	☐ Good ☐ Fair ☐ Critical
Presence of Zoonotic Risks for Humans (e.g., fleas, mange, bites)	☐ None observed ☐ Possible ☐ Confirmed → Specify: ________
Intervention Priority	☐ High ☐ Medium ☐ Low
Final Notes	

Veterinarian Name ____________________________

Date ____________________________

Signature ____________________________
