# Supplementary material for: Development of an Operational Protocol for Animal Hoarding: A Conceptual Proposal Based on Multidisciplinary Field Experience
Source: Animals (Basel). 2025 Nov 6;15(21):3222. doi: 10.3390/ani15213222 (PMC12610984; doi:10.3390/ani15213222)
Supplement: Supplementary file 1 [file animals-15-03222-s001.zip › S4-simulated case - POF.pdf]

PRELIMINARY OBSERVATIONAL FORM – Animal Hoarding Context

Simulated Case "Maria"

Operational Version for Public Veterinarians and Staff from Other Agencies
Estimated completion time: 10–15 minutes

- Date: 2025-06-09
- Operator: Dr. A. (fictitious)
- Agency: Local Veterinary Health Authority
- Inspection address: Via Vittoria 8, Rome, Italy

1. Type of Premises and Housing Conditions

☐ Private home ☑ Apartment ☐ Farm structure ☐ Other: ___________
Housing accessibility:
☑ Easily accessible ☐ Difficult access ☐ Unsafe / hazardous

2. Resident's name (if available): Maria

Approximate age: 67
Access allowed?
☐ Yes ☑ Partial ☐ No
Cooperation level:
☐ Friendly ☑ Neutral ☐ Evasive ☐ Hostile

3. Animals Present (visual estimate or as declared)

Please fill in even in case of approximate estimates.

**Species**	**Estimated No.**	**Puppies/Kittens?**	**Active reproduction?**	**Visible signs of distress?**
Dogs	2	☐ Yes ☑ No	☑ Yes ☐ No	☑ Yes ☐ No
Cats	28	☑ Yes ☐ No	☑ Yes ☐ No	☑ Yes ☐ No
Other (specify): ______	______	☐ Yes ☐ No	☐ Yes ☐ No	☐ Yes ☐ No

Notes:
Unneutered cats, signs of parasitosis and undernourishment. Dogs with dermatological and behavioral issues.

4. Fragile Individuals in the Household

☐ None
☐ Minors → Age: ______ Number: ______ Involvement: ▫ direct ▫ indirect
☐ Elderly individuals (>75 years) → Number: ______
☑ Persons with disabilities or mental fragility → Specify: *Previous diagnosis of depression*

5. Human–Animal Relationship

☑ Affective (talks to animals, pets them, shows emotional bonding)
☐ Neglectful (lacks care, does not provide food or hygiene)
☑ Ambivalent (alternating affection and neglect; emotional disorganization)
☐ Instrumental (uses animals for external purposes: begging, control, imposed companionship)
☐ Other: ____

6. Environmental Conditions (direct observation)

**Indicator**	**Observed level**	**Notes**
Strong odour of feces/urine	☑ Yes ☐ No ☐ Not assessable	Strong ammonia odour
Visible presence of feces	☑ Yes ☐ No ☐ Not assessable	All rooms affected
Object accumulation / clutter	☐ None ☐ Moderate ☑ Severe	Hazardous clutter
Fire/fall hazard	☑ Present ☐ Absent	Electrical overload and blocked exits
Ventilation / natural light	☐ Adequate ☑ Inadequate	Closed shutters, high humidity

Signs of infestation (insects/rodents)	☑ Yes ☐ No ☐ Not assessable	Presence of insects, fleas, signs of rodents

---

7. Additional Observations (Optional)

☑ Hoarding of objects or waste
☑ Unsafe structures or electrical systems
☐ Presence of carcasses or animal remains
☑ Rodents or pests
☐ Other: ____

8. Subject's Behavioral Indications

Open-ended questions (verbatim):

- *"How do you feel about your current situation with the animals?"*

"They are my only family now. They all need me."

- *"What is the hardest thing to manage right now?"*

"Cleaning everything by myself... it's overwhelming."

- *"If you could change something, what would it be?"*

"Maybe just someone to help me a bit, but I can't let them go."

- *"Have you ever received help for this situation?"*

"They once took some cats, but it was terrible."

Problem awareness:
☐ Aware ☑ Minimizes ☐ Denies ☐ Not assessable

Collaboration during the interview:
☐ Friendly ☑ Formal ☐ Avoidant ☐ Hostile

Operator's behavioral notes:
Polite and articulate, but emotionally rigid. Repeatedly avoids direct questions about hygiene. Contradictory answers regarding past interventions.

9. Preliminary Risk Assessment

Overall Risk Level:
☐ 1 – No risk
☐ 2 – Mild risk
☐ 3 – Moderate risk
☐ 4 – High risk
☑ 5 – Critical risk

Justification for the assigned score:
Severe unsanitary conditions, high number of animals, confirmed reproductive activity, previous failed intervention, lack of clinical follow-up, and resident's fragile psychological condition.

10. Recommendations and Referrals

Suggested actions (check all applicable):
☐ Periodic follow-up (within 30 days)
☑ Multidisciplinary team activation
☑ Psychosocial evaluation (social/mental health services)
☑ Comprehensive veterinary assessment (→ Veterinary Health Form)
☑ Emergency action / Judicial authority notification

Additional notes:
Immediate coordination with social and mental health services advised. Veterinary assessment required for urgent triage and potential rehoming strategy. Judicial oversight may be necessary due to recurring complaints and prior failed intervention.

Operator's Full Name: Dr. A. (fictitious)
Affiliated Service/Agency: Local Veterinary Health Authority
Date: 2025-06-09 **Signature:** ____________________________
