# Supplementary material for: Development of an Operational Protocol for Animal Hoarding: A Conceptual Proposal Based on Multidisciplinary Field Experience
Source: Animals (Basel). 2025 Nov 6;15(21):3222. doi: 10.3390/ani15213222 (PMC12610984; doi:10.3390/ani15213222)
Supplement: Supplementary file 1 [file animals-15-03222-s001.zip › S5- simulated case - ICRAH.pdf]

STRUCTURED CLINICAL-RELATIONAL INTERVIEW FOR CASES OF ANIMAL HOARDING (ICRAH)
Simulated case "Maria"

SECTION 1 – PERSONAL DATA AND SOCIAL RELATIONSHIPS

The collection of personal data must be conducted respectfully and in proportion to the context. In cases of significant discomfort or refusal, the interview may proceed without gathering the full set of information.

1.1 Identity and Contact Information

"How would you prefer to be addressed?"
"Maria is fine."
"Is there anything you would like to share to help us get to know you better (e.g., age, identity, preferred pronouns)?"
"I'm 67, I live alone. I don't need much else."
(Only if necessary): "May we record your name and contact information for possible future follow-up?"
"If it's necessary, yes, but I don't like being called too often."

Full Name: Maria
Address: Via Vittoria 8, Rome, Italy
Contact details (phone/email): Not disclosed
Age: 67
Gender: ☐ Male ☑ Female ☐ Other ☐ Prefer not to say
Additional notes (name use, preferences, etc.): Prefers to be addressed by first name only; sensitive to repeated contacts.

"May we see an identity document and record the relevant details?"
"I'd rather not. I had bad experiences with people using my data."

Type of document: ☐ Identity card ☐ Driver's license ☐ Passport ☐ Other: __________
Document number/ID: Not provided

1.2 Employment Status and Educational Background

Questions:
"What kind of activity are you currently involved in, if any?"
"I'm retired now. I used to work as a school secretary for over 30 years."
"Could you tell me about your educational background?"
"I finished high school, but never went to university. Back then it wasn't common for girls."
Observational Notes:
The subject appears composed and open when discussing her past job. Speaks with pride but some nostalgia. Educational background seems coherent with age and cultural context.

1.3 Economic Condition

Question:
"Could you tell me how you perceive your current financial situation? Are you able to cover daily expenses, including those related to your own needs and the animals?"
"I receive a pension and some help from my niece. I manage, but when there's a vet emergency, it's hard."

Checklist – Signs of Economic Stability or Vulnerability:
☐ Sufficient and stable income
☑ Irregular or variable income
☑ Reliance on external aid (family, services, volunteers)
☑ Difficulty covering daily expenses
☑ Difficulty covering veterinary costs
☐ Refusal or discomfort in discussing financial matters
☐ Other: ______________________________
Observational Notes:
Subject talks openly, without shame, but displays mild signs of concern. Emotional tone calm yet with visible tension when discussing animal-related expenses.

1.4 Co-habitation and Family Context
Questions:
"Do you currently live with someone?"
"No, I live alone. Just me and the animals."
"Are there people who care for you or whom you care for?"
"My niece comes by sometimes, but I mostly take care of myself."
Checklist – Presence of Co-habitants and Contextual Fragilities:
☑ Lives alone
☐ Lives with family / friends / partner
☐ Positive relationships with co-habitants
☐ Family conflict or relational difficulties
☐ Presence of minors
☐ Presence of elderly or disabled individuals
☐ Other relevant elements: ___________________________
Observational Notes:
The subject's living alone is clearly a factor of vulnerability, though she does not express feelings of abandonment. Emotional tone suggests self-sufficiency, but mild isolation.

1.5 Personal History
Questions:
"Are there any moments in your life that you consider particularly important and that might help me better understand your current situation?"
"I lost my husband 12 years ago. After that, the animals became everything to me."
"Have you experienced events that have left a deep mark on you?"
"Yes, losing him. And also my mother, many years before. I've always dealt with grief by staying active and caring for others."
"Is there anything in your personal history that you feel the need to share with me?"
"Not really. I just feel I have a duty to protect lives others throw away."
Checklist – Significant Events or Emerging Vulnerabilities:
☑ Presence of traumatic events or recent losses
☑ Isolation, separations, bereavement, or abandonment
☑ Positive experiences of coping or resilience
☐ Complex or unstable relational history
☑ Coherent and reflective narrative

☐ Difficulty discussing personal history / tendency to avoid
☐ Other: ______________________________
Observational Notes:
The subject shows capacity for reflection and narrative coherence. Emotional tone is contained but authentic. The loss of significant relationships appears to have shaped the current animal-human bond.

1.6 Health and Daily Habits
Open-ended questions
"Are there any aspects of your health you'd like to talk about, that you feel are important for understanding your current well-being?"
"Well… I'm not as strong as I used to be. I get tired quickly. My doctor says it's just age, but I've had some difficult years."
"Are you currently undergoing treatment, taking prescribed medications, or receiving any kind of healthcare support?"
"Yes, I take something for anxiety and to sleep. And some pills for my blood pressure. But I manage."
"Are there habits that you feel contribute positively to your well-being, or conversely, challenges you face in daily life?"
"Feeding my cats keeps me going. But cleaning is hard. I can't do it all anymore."

Qualitative Indicators – Relevant Medical or Behavioral Information
☑ Known medical diagnoses
☑ Regular use of prescribed medications
☑ Ongoing treatments or therapies
☐ Substance use or abuse (alcohol, medications, other)
☑ Health conditions that may interfere with daily functioning
☐ No relevant health conditions reported
☐ Other: ______________________________
Observational Notes:
Subject appears frail but lucid. She expresses concern over fatigue and mobility, especially regarding housework. Mentions anxiety and sleep disturbances. Narration is partially coherent, occasionally vague. Appears autonomous but overwhelmed by daily tasks.

1.7 Social Network and Relationships
Open-ended questions
"Are there people you feel you can rely on, even just to talk to or ask for help if needed?"
"Not really. I used to talk to a neighbor, but she moved. Now it's just me and the animals."
"Do you maintain regular contact with family members, friends, or neighbors? Do you enjoy receiving visitors?"
"No family left. I don't really like people coming over. The cats get scared."
"Do you feel supported by your social network, or do you sometimes feel alone?"
"Most of the time I feel alone. But I don't mind. I have my cats."
Qualitative Indicators – Presence of Supportive Relationships
☐ Stable presence of family or friendship bonds
☐ Active and perceived supportive social network

☐ Occasional but meaningful relationships
☑ Perceived social isolation
☑ Limited emotional or practical support
☐ Conflicted or interrupted relationships
☐ Other: ____________________________

Observational Notes:
The subject expresses emotional detachment from others, does not mention any current relationships. Tone is flat, resignation is evident. Protective elements are limited to the perceived emotional bond with animals.

1.8 Previous Experiences with Authorities or Services
Open-ended questions
"Has anyone—such as neighbors, social or health services, or authorities—ever pointed out anything to you regarding your animals or your home?"
"Yes. A few years ago someone came and took some of my cats. They said it was for their good, but they never came back to tell me what happened."
"How did you experience these situations? Did they seem unfair, or did they give you something to reflect on?"
"It felt like a punishment. No one asked me how I was. I felt betrayed."
"Has anyone ever offered help? If so, how did you feel in that moment?"
"Not real help. Just judgment. They wanted to take the animals, not help me."
Qualitative Indicators – Past Experiences and Attitude Toward Services
☐ No prior experiences with services
☐ Previous positive or collaborative experiences
☑ Conflictual or perceived invasive experiences
☐ Collaborative attitude toward potential interventions
☑ Distrustful or closed attitude toward services
☐ Other: ____________________________

Observational Notes:
The subject appears guarded and defensive when recalling past events. Emotional tone is marked by bitterness and distrust. She avoids using the word "authorities" and tends to portray herself as the only one caring for the animals. Defensive posture throughout the conversation.

1.9 Recent Critical Events
Open-ended question
"In the past few months, have there been any difficult events or situations that have changed something in your daily life or in your relationship with your animals?"
"Yes. My health got worse and the cleaning got out of hand. I lost a cat too. I don't even know why—he just stopped eating."
Checklist – Signs of Risk or Well-being
☐ No relevant events reported
☐ Isolated but managed events (e.g., processed bereavement)
☑ Serious or repeated events with clear impact on well-being and daily management
☐ Other: ____________________________

Observational Notes:
Maria becomes visibly emotional when mentioning the loss of the cat. Her voice softens and

she looks away. Signs of guilt and helplessness emerge. There's a clear overlap between personal deterioration and decline in animal care.

1.10 Relationships with Neighbors
Open-ended question
"What is your relationship like with your neighbors, particularly regarding the presence of animals? Have there ever been comments, complaints, or unpleasant situations?"
"They don't say anything to my face, but I know they talk. Someone must have called the authorities last time. I just try to keep to myself."
Checklist – Signs of Risk or Well-being
☐ Cooperative and peaceful relationship with neighbors
☐ Fragile tolerance or occasional complaints
☑ Ongoing conflicts, reports, repeated tensions
☐ Other: ____________________________
Observational Notes:
She displays signs of tension and mistrust when speaking about the neighbors. Uses vague references like "they talk" and "someone called," suggesting hypervigilance. No active conflict described, but social withdrawal is evident.

SECTION 2 – ANIMALS AND MANAGEMENT

2.1 Current Presence of Animals
Open-ended questions
"How many animals are currently living with you?"
"I have two dogs and… I think about 28 cats. Maybe more, some hide during the day."
"What kind are they (dogs, cats, others)? How would you describe them (age, origin, particular traits)?"
"They're all rescues, you know? Some I found on the street, others were born here. Most of the cats are mixed, I don't know the age. A few are older and need more care."
"How do you organize their daily care?"
"I feed them in the morning and at night. I try to clean the litter boxes when I can. The dogs go out in the yard downstairs with me, but not every day."
Checklist – Animal Presence and Management Capacity
☐ Number of animals proportionate to available resources and space
☐ High number of animals, but managed with commitment
☑ Excessive number relative to caregiving capacity
☐ Presence of species that are incompatible with each other
☑ Difficulty providing a complete or updated description
Observational Notes:
Maria shows emotional involvement but struggles to quantify the animals or describe their needs individually. Some confusion in estimating the actual number. Care appears uneven. Strong bond, but evident signs of overload.

2.2 Origin and Arrival of the Animals
Open-ended questions
"How did these animals come to live with you? Did you seek them out, or did they arrive spontaneously?"

"Some I found in the trash bins. Others just showed up and I couldn't turn them away. People know I don't say no."

"Do you continue to take in new animals? If so, what motivates you to do so?"

"If they need help, what should I do? Leave them outside to die? I can't."

Checklist – Arrival Modalities and Predominant Motivation

☐ Occasional and purposeful intake (e.g., adoption, rescue)
☐ Frequent, not always planned arrivals
☑ Accumulation due to repeated rescues or abandonment cases
☑ Tendency to assume responsibility without prior evaluation
☐ Lack of awareness regarding the motivations for intake

Observational Notes:

Maria exhibits strong rescuing motives, with poor boundaries in accepting new animals. Emotional tone alternates between pride and guilt. Clear difficulty setting limits. Motivations are protective but impulsive.

2.3 Daily Management Practices

Open-ended questions

"How is your day structured with your animals?"

"They come first. I wake up and feed them. Then I clean a bit. I take the dogs down if I feel ok. Sometimes I skip meals myself."

"Are there specific times dedicated to their cleaning, feeding, or medical care?"

"Feeding is always done. Cleaning… I do what I can. I don't have much strength anymore. Medicines, only if I have some left."

"Do you feel able to manage everything, or are there difficulties?"

"It's hard, yes. I wish I could do more. I feel like I'm always behind."

Checklist – Observable Indicators of Daily Management

☐ Regular and structured caregiving routine
☑ Care is provided, but with some logistical or physical challenges
☑ Disorganized management, partial or neglected care
☐ Delegation to others or presence of external help
☐ Lack of awareness of the animals' basic needs

Observational Notes:

Narrative suggests emotional dedication but lack of physical and organizational capacity. Some inconsistencies between reported practices and direct observation. Equipment is insufficient, litter boxes overcrowded. Maria shows signs of fatigue and helplessness but continues to prioritize animal care.

2.4 Hygiene and Living Environment of the Animals

Open-ended questions

"Where do the animals primarily live? Are there specific areas designated for them?"

"They're mostly inside the apartment. The cats go wherever they want… the bedroom, the kitchen. I try to keep them out of the bathroom but it's not always possible."

"How is the cleaning of their living areas organized?"

"I clean as much as I can… I change the litter when I have new sand. But there's always someone who pees outside the box."

"Is there anything you would like to improve, or that concerns you in particular?"

"Yes… the smell. Sometimes I open the windows but it's not enough. I'd like to do better but I'm tired all the time."

Checklist – Observations on the Animals' Living Environment

☐ Clean, well-ventilated spaces with appropriate materials
☑ Functional environments, but showing signs of caregiver fatigue
☑ Overcrowded areas, strong odors, presence of feces/urine
☑ Confined animals, closed or restricted-access spaces
☐ Inappropriate or hazardous areas (e.g., mold, sharp objects, escape risk)

Observational Notes:

Overwhelming smell of urine and feces, especially in the hallway. Multiple litter boxes visible but many are soiled. Food and water bowls mixed together, often dirty. Ventilation insufficient. Cats were seen hiding under furniture or clustering near windows. Dogs appear calmer but confined in a small area.

2.5 Behaviors and Relationships Among Animals

Open-ended questions

"How do your animals usually behave with one another?"

"They're used to each other. Some cats fight, but it's like family squabbles."

"Have you ever noticed signs of tension, aggression, or unusual behaviors?"

"One of the males used to attack the others… I had to separate him. And some just stay in corners. They're shy."

"How would you describe your relationship with them?"

"They're my life. They wait for me, they listen. I feel understood when I'm with them."

"Can you tell if there are affectionate bonds among the animals, or if some appear excluded or isolated?"

"There are little groups… the siblings sleep together. A few keep to themselves, but that's just their nature."

Checklist – Indicators of Intra-/Inter-species Relational Dynamics

☑ Harmonious interactions among animals
☑ Occasional conflicts or tensions
☐ Frequent or marked conflict among specific animals
☑ Balanced relationship between person and animals
☑ Signs of overinvolvement or emotional symbiosis
☑ Atypical behaviors (stereotypies, avoidance, excessive vocalizations)
☑ Presence of animals who are isolated, passive, or withdrawn

Observational Notes:

Some cats exhibit hiding behavior or avoid contact. A few display repetitive pacing. Dogs appear calmer but stay close to the resident. Maria displays intense emotional involvement, often anthropomorphizing the animals. Her tone softens when naming individual animals, with visible emotional attachment.

2.6 Management of Animal Death

Open-ended question

"When one of your animals dies, what usually happens? How do you cope with that moment? What do you choose to do with the body?"

"I wrap them in a towel and keep them with me for a while. I need to say goodbye. If I can, I bury them at my friend's garden. Once I kept one for a few days in the freezer."

Checklist – Indicators in the Management of Animal Loss
☐ Proper disposal through veterinary services or legal authorities
☑ Burial at home, in a garden or rural area
☑ Retention of the animal's body in the home (e.g., mummification, freezing, taxidermy, other)
☑ Difficulty or refusal to confront the loss
☑ Ritualized or idealized narrative of death
☑ Intense emotional reaction to the loss (e.g., crisis, social withdrawal, increase in accumulation)
☐ Other (specify): ________________
Observational Notes:
Maria becomes visibly emotional while recalling animal deaths. Her narrative includes spiritual references (e.g., "they wait for me somewhere") and a need to physically hold the body. Reactions suggest unresolved grief and difficulty separating from the animal. Attachment is described as unique and irreplaceable for each pet.

SECTION 3 – LIVING CONDITIONS AND ENVIRONMENTAL ASPECTS

3.1 General Hygiene Conditions
1. How would you describe the environment where you live with your animals? Are there designated areas for them, or do you share all spaces?
"We live all together. They go everywhere, even on the bed and couch. It's their home too."
2. Have you ever had difficulty keeping the home clean or tidy? Are there times when it becomes particularly hard to manage?
"Yes… especially when I'm not feeling well. Cleaning takes so much energy, and it feels like I can never finish."
3. Are there rooms or areas in the house that you avoid using or that have become difficult to maintain?
"The second bedroom is full of things. I haven't been in there for a while. It's just for storage now."
4. Have you had any problems with the structure of the house (e.g., dampness, plumbing, mold, damage)?
"Yes, there's a leak in the bathroom ceiling. And sometimes the kitchen sink gets clogged."
5. Are the windows used regularly? Is there adequate air circulation and natural light in the home?
"I try to open them, but not too much. The cats might escape. I prefer to keep the curtains closed too."
6. Have you ever noticed the presence of pests, such as insects, rodents, or persistent odors?
"Sometimes there are flies. And yes, it smells—especially in summer."
7. Have you observed any accumulation of objects, decomposing materials, or hard-to-remove waste?
"Some stuff piles up. I keep meaning to throw things out but… I never have the time or energy."
Checklist – Indicators of Environmental Risk or Well-being
☐ Organized and accessible living spaces
☐ Clean environment with regular hygiene management
☑ Difficulty maintaining cleanliness or order
☑ Unusable or overcrowded areas
☑ Presence of mold, structural damage, or hazards (e.g., exposed wiring or pipes)
☑ Poor air circulation or dark indoor environments
☑ Strong or persistent odors

☑ Presence of insects, rodents, or other pests
☑ Presence of decomposing organic materials or accumulated waste
☐ Other: ____________________________

Observational Notes:
The home shows signs of environmental degradation: cluttered hallways, strong odor of urine and feces, accumulation of items in corners. One room is completely inaccessible due to stored materials. Windows are closed or covered, limiting light and ventilation. Evidence of flies and damp spots on ceilings noted. Subject expresses emotional resignation but also a sense of sharing space equally with the animals, indicating limited awareness of the environmental risks.

SECTION 4 – PSYCHOLOGICAL ASPECTS AND MOTIVATIONS

4.1 Emotional Bond and Perceived Role of Animals

1. How would you describe the emotional bond you have with your animals?
"They are everything to me. I don't know what I'd do without them."
2. What role do they play in your life?
"They give me company, peace. They're my family now."
3. Do you believe that their presence fulfills a specific personal need?
"Yes… I feel less alone. They're the only ones who don't judge me."
4. Have you ever felt that your animals understand you better than people do?
"Absolutely. They feel when I'm down. They always stay close."
5. Have your animals helped you through difficult moments in your life?
"Yes, especially after I lost my sister. Without them, I don't think I'd still be here."
6. Have you ever thought about being separated from any of them? What emotions did that provoke?
"No. Just thinking about it makes me panic. They need me. And I need them."
7. Do you think someone else could take care of them? What are your thoughts on that?
"No one could love them like I do. They'd feel abandoned."

Checklist – Emotional Role and Function of the Animals
☐ Balanced emotional bond, with awareness and openness
☐ Presence of emotional needs compensated by animals, with some degree of reflection
☑ Overwhelming bond, with strong substitutive or regulatory function; significant dependency
☑ Rejection of the idea of separation or delegation
☐ Other: ____

Observational Notes:
Maria speaks of the animals in deeply affective and exclusive terms. Her tone is affectionate but reveals a strong emotional dependency. Shows resistance and distress at the idea of separation. High idealization of the bond; the animals are described as irreplaceable and emotionally rescuing.

4.2 Social Perception and Awareness of the Problem

8. Has anyone ever pointed out that the number of animals you keep might be too high? How did you respond?
"Yes, a couple of neighbors complained once. But they don't understand. The animals are fine."
9. In your opinion, what are the main challenges involved in managing all these animals?
"There's a lot to do—cleaning, feeding, the expenses... but I manage. It's just tiring sometimes."

Checklist – Social Awareness
☐ Full awareness and openness to dialogue

☑ Defensive reactions, but partial acknowledgment
☐ Denial of the problem or refusal to engage in discussion
☐ Other: ____
Observational Notes:
Maria displays partial insight into the difficulties of management, but frames complaints as lack of understanding by others. Shows mild defensiveness but does not completely reject the idea of difficulty. Emotional tone somewhat tense when addressing external criticism.

4.3 Impulsivity and Capacity for Letting Go
10. How do you experience the desire or impulse to take in new animals or objects?
"It's hard to say no. If I see one in need, I just can't leave it there."
11. Would it be difficult for you to part with some of your animals? In what way?
"Unthinkable. It would feel like losing a part of me. I wouldn't sleep at night."
Checklist – Impulsivity and Attachment
☐ Good capacity for self-control and separation
☐ Moderate discomfort, occasional impulses
☑ Intense distress or inability to separate, uncontrolled intake behavior
☐ Other: ____
Observational Notes:
The subject reveals compulsive intake behavior and marked difficulty in setting boundaries. Expresses intense distress at the idea of separation. No evidence of filtering or delaying the decision to adopt or rescue. Emotional overinvolvement appears to drive decisions more than practical evaluation.

4.4 Underlying Motivations and General Emotional State
12. What has motivated you to take in so many animals?
"They needed me. And I needed them too. I've always been the one who fixes things."
13. How would you describe your general emotional state in recent times?
"I feel tired… sometimes very alone. But with them, I feel useful again."
14. Do you feel lonely or isolated? Do you find it difficult to maintain meaningful relationships?
"Yes… people are hard. I don't go out much anymore."
15. Would you consider seeking professional support to improve your situation?
"I'm not sure. I wouldn't want them to take the animals away. But… maybe if I could trust them."
Checklist – Motivations and Openness to Support
☐ Clear motivations, stable emotional state, openness to support
☐ Partial motivations, unstable emotional state, limited openness
☑ Confused or denied motivations, compromised emotional state, unwillingness to engage
☐ Other: ____
Observational Notes:
Maria's motivation is emotionally driven and idealized, with signs of psychological fragility. Displays ambivalence toward help: fears losing the animals, but also reveals exhaustion and isolation. Emotional state appears compromised; tendency toward social withdrawal and low trust in external support.

SECTION 5 – RISK ASSESSMENT AND WILLINGNESS TO COLLABORATE

5.1 Perception of the Situation and Risk Awareness

1. If you had to describe your current situation with your animals, what would you say?
"They're my life. I do everything for them. Things could be tidier, but nothing serious."
2. Do you feel everything is under control, or are there aspects that are sometimes challenging?
"Sometimes it gets tiring, especially when I'm not feeling well. But I manage."
3. Do you think your animals are doing well? And how do you feel?
"Yes, they're happy. I get tired, but I feel fulfilled."
Checklist – Indicators of Awareness
☐ Realistic perception of the situation
☑ Partial or ambivalent awareness
☐ Denial or underestimation of the issues
Observational Notes:
Maria acknowledges fatigue but tends to minimize the environmental and health-related challenges. Shows emotional attachment and subjective fulfillment but lacks full awareness of objective risk factors.

5.2 Willingness to Change and Collaborate
4. Have you ever thought that, with a bit of help, some things could improve?
"Maybe, yes… but I don't want people to come and judge me."
5. What do you think about the possibility of gradually reducing the number of animals?
"I couldn't do that. They've been with me for so long."
6. If you have tried to change something in the past, what happened?
"I tried to let go of one once, and I couldn't sleep for days."
Checklist – Indicators of Openness
☐ Willingness to engage and consider habit changes
☑ Ambivalence or uncertainty about change
☐ Rigidity and refusal of any change
Observational Notes:
Subject exhibits emotional resistance to change, but there is a subtle recognition of the burden. Expresses fear of judgment and distress at separation. A therapeutic alliance may need time and gradual trust-building.

5.3 Previous Experiences and Future Planning
7. Have you previously received complaints or had issues with others regarding your animal care?
"Yes, but it was exaggerated. People don't get how much I care."
8. In an ideal scenario, how many animals do you think would be appropriate to have?
"I never thought about a number. If they need me, I help."
9. What would you like to change in order to feel better—both for yourself and for them?
"Maybe more space, more help with cleaning. But I don't want to give anyone away."
Checklist – Indicators of Future Planning
☐ Integrated past experiences and constructive outlook
☑ Partially processed conflicts or difficulties
☐ Rejection of the past and lack of future perspective
Observational Notes:
Maria shows partial processing of past conflict, tends to justify her behavior, and lacks a concrete future plan. Her narrative reflects protective intentions but poor strategic planning or self-care focus.

5.4 Available Resources and Interdisciplinary Collaboration
10. What types of support would be most helpful to you right now?
"Maybe help with cleaning or with food… but nothing that involves taking them away."
11. In your opinion, who could support you along this path?
"I trust my vet. And maybe someone kind who doesn't judge."
12. Would you be willing to collaborate with multiple professionals, such as a veterinarian, psychologist, or social worker?
"Only if I can keep the animals and feel respected."
Checklist – Possible Resources and Alliances
☑ Clearly identified needs, openness to networking
☐ Vague needs, limited willingness
☐ Rejection of external help, unwillingness to collaborate
☐ Existing collaboration with professionals (specify): __________
Observational Notes:
Maria expresses conditional openness to collaboration, grounded in trust and emotional safety. Identifies concrete needs and mentions the veterinarian as a potential ally. Emphasizes respect and autonomy.

SECTION 6 – NARRATIVE SUMMARY AND METAPHOR
Open-ended question for the individual
"If you had to describe your relationship with your animals using an image or a metaphor, what would it be?"
"Like a little boat in the storm… they're the ones who keep it from sinking."
Clinical-Relational Summary by the Practitioner
Maria, a 67-year-old woman, shows a deeply emotional and symbiotic attachment to her animals, who she perceives as her only source of comfort and identity. She demonstrates partial awareness of her challenges and expresses ambivalence toward external support. Although environmental risks and emotional overload are present, her narrative reveals preserved empathic abilities and a desire not to be judged. The metaphor used captures both the fragility and central role of the animals in her life. A gradual, respectful multidisciplinary approach is recommended, starting with her trusted veterinarian and focusing on emotional safety, practical assistance, and slow trust-building.

Operator's Full Name: Dr. A. (fictitious)

Affiliated Service/Agency: Local Veterinary Health Authority

Date: 2025-06-09 **Signature:** ____________________________
