# Supplementary material for: Development of an Operational Protocol for Animal Hoarding: A Conceptual Proposal Based on Multidisciplinary Field Experience
Source: Animals (Basel). 2025 Nov 6;15(21):3222. doi: 10.3390/ani15213222 (PMC12610984; doi:10.3390/ani15213222)
Supplement: Supplementary file 1 [file animals-15-03222-s001.zip › S6-simulated case VHR.pdf]

VETERINARY HEALTH FORM FOR ANIMALS IN HOARDING CONTEXTS (VHR)

Simulated case "Maria"

Animal Owner Maria

**Section**	**Details**
**1. General Information about the Animal**	
Animal ID	NICO-01
Species	Cat
Breed	Domestic Longhair
Estimated Age	9 years
Sex	☒ Male ☐ Female ☐ Not Determinable
Reproductive Status	☐ Intact ☒ Spayed/Neutered ☐ Not Detected
Microchip	☒ Yes (Number: 981000123456789) ☐ No ☐ Not Detected
**2. General Physical Conditions**	
Weight	4.2 kg (☒ measured / ☐ estimated)
Body Condition Score (BCS)	☐ 1 (severely underweight) ☐ 2 (underweight) ☒ 3 (normal) ☐ 4 (overweight) ☐ 5 (obese)
Coat Condition	☒ Clean ☐ Dirty ☐ Matted/Tangled ☐ Alopecia
Skin Conditions	☒ Normal ☐ Lesions ☐ Parasites ☐ Specify: __________
Eye Conditions	☐ Normal ☒ Reddened ☐ Discharge ☐ Type: __________
Ear Conditions	☒ Normal ☐ Dirty ☐ Infections ☐ Specify: __________
**3. General Health Status**	
Signs of Dehydration	☒ Absent ☐ Mild ☐ Moderate ☐ Severe
Signs of Malnutrition	☒ Absent ☐ Present ☐ Specify: __________
Dental/Oral Problems	☐ Absent ☒ Present ☐ Description: Mild tartar accumulation
Mobility	☒ Normal ☐ Lameness ☐ Muscle Atrophy ☐ Specify: __________
Evident Pain Signs	☒ Absent ☐ Present ☐ Location: __________
Clinical Signs of Disease	☐ Cough ☐ Vomiting ☐ Diarrhea ☒ Other: Mild conjunctivitis
Zoonotic Risks Identified (e.g., fleas, mange, bites)	☒ No ☐ Yes → Specify: __________________________
**4. Behavioral Status**	
Behavior Toward Humans	☒ Friendly ☐ Fearful ☐ Aggressive ☐ Other: __________
Behavior Toward Other Animals	☒ Social ☐ Fearful ☐ Aggressive ☐ Other: __________
Stereotypies or Repetitive Behaviors	☒ Absent ☐ Present ☐ Type: __________
**5. Preliminary Diagnosis and Notes**	
Diagnosis	Mild conjunctivitis and dental tartar. General health stable.
Recommended Examinations	Full dental check-up; ocular swab if conjunctivitis persists.

Suggested Therapies	Dental hygiene treatment; saline eye drops twice daily for 5–7 days. Monitor symptoms.
Additional Notes	The cat appears well integrated and displays normal behavior patterns. Living in a calm indoor environment. No signs of stress or distress.
**6. Photographic Documentation**	
Photo 1	Full body Date: 2025-06-09
Photo 2	Lesion detail (eye) Date: 2025-06-09
**7. Welfare Assessment (AWIN Protocol)**	
Access to Food and Water	☒ Adequate ☐ Inadequate
Physical Comfort	☒ Normal ☐ Limited ☐ Other: __________
Overall Health Status	☒ Good ☐ Compromised ☐ Other: __________
Normal Behaviors	☒ Present ☐ Absent ☐ Other: __________
Social Interaction	☒ Adequate ☐ Evident Problems ☐ Other: __________
Response to Stimuli	☒ Normal ☐ Altered ☐ Other: __________
Exploratory Behavior	☒ Present ☐ Absent ☐ Other: __________
**8. Final Evaluation**	
Overall Welfare Status	☒ Good ☐ Fair ☐ Critical
Presence of Zoonotic Risks for Humans (e.g., fleas, mange, bites)	☒ None observed ☐ Possible ☐ Confirmed → Specify: ________
Intervention Priority	☐ High ☐ Medium ☒ Low
Final Notes	No intervention needed beyond preventive care. The animal is in a stable condition. Nicoletta appears able and motivated to care for this cat appropriately.
Veterinarian Name	Dr. A.
Date	2025-06-09
Signature	____________________________
